# Deletion of Tgf‐β1 From CD206^+^ M2 Macrophages Ameliorates Obesity‐Induced Suppression of Myogenesis and AMPK Phosphorylation in Skeletal Muscle

**DOI:** 10.1002/jcsm.70322

**Published:** 2026-06-18

**Authors:** Muhammad Bilal, Le Duc Anh, Nguyen Quynh Phuong, Sana Khalid, Allah Nawaz, Memoona, Muhammad Rahil Aslam, Tomonobu Kado, Yoshiyuki Watanabe, Ayumi Nishimura, Yoshiko Igarashi, Aamir Sharif, Yasuhiro Onogi, Tsutomu Wada, Ryuji Hayashi, Kenichi Hirabayashi, Seiji Yamamoto, Takashi Nakagawa, Hisashi Mori, Isao Usui, Masaru Kato, Shiho Fujisaka, Kazuyuki Tobe

**Affiliations:** ^1^ First Department of Internal Medicine, Faculty of Medicine University of Toyama Toyama Japan; ^2^ Research Center for Pre‐Disease Science, Faculty of Education and Research Promotion University of Toyama Toyama Japan; ^3^ Advanced Postdoctoral Fellowships of the Japan Diabetes Society (JDS) Tokyo Japan; ^4^ Stem Cell Center and Tissue Bank Phenikaa University Hospital Hanoi Vietnam; ^5^ Clinical Oncology, Faculty of Medicine University of Toyama Toyama Japan; ^6^ Department of Molecular Neuroscience, Faculty of Medicine University of Toyama Toyama Japan; ^7^ Section of Integrative Physiology and Metabolism Joslin Diabetes Center, Harvard Medical School Boston Massachusetts USA; ^8^ Department of Neurophysiology, Faculty of Medicine University of Yamanashi Yamanashi Japan; ^9^ Department of Clinical Pharmacology, Faculty of Pharmaceutical Sciences University of Toyama Toyama Japan; ^10^ Department of Diagnostic Pathology, Faculty of Medicine University of Toyama Toyama Japan; ^11^ Department of Pathology, Faculty of Medicine University of Toyama Toyama Japan; ^12^ Department of Molecular and Medical Pharmacology, Faculty of Medicine University of Toyama Toyama Japan; ^13^ Department of Endocrinology and Metabolism Dokkyo Medical University Tochigi Japan; ^14^ Faculty of Medicine University of Toyama Toyama Japan

**Keywords:** M2 macrophage‐derived Tgf‐β1, mitochondrial function, muscle dysfunction, sarcopenic obesity

## Abstract

**Backgrounds:**

Obesity and diabetes impair the ability of the muscle to regenerate, repair and remodel, resulting in a gradual decrease in muscle mass and function. However, the underlying mechanisms and effective therapeutic strategies remain poorly understood. M2 macrophages within skeletal muscle play an important role in tissue recovery following injury. This study aims to investigate the role of M2 macrophages derived transforming growth factor‐beta 1 (Tgf‐β1) in regulation of skeletal muscle function under diet‐induced obese conditions.

**Methods:**

An CD206^+^ M2 macrophage‐specific Tgf‐β1 gene knockout (Tgf‐β1 KO) mouse model was generated by crossing CD206‐CreER^T2^ mice with Tgf‐β1^f/f^ mice, followed by tamoxifen administration to induce Tgf‐β1 gene deletion. Mice were then placed on a high‐fat diet (HFD) for 12 weeks to develop obesity‐induced skeletal muscle dysfunction. The study used multiple physiological and molecular analyses, including exercise tolerance, hanging time, grip strength, glucose and insulin tolerance tests, western blotting, RT‐qPCR and others.

**Results:**

The present findings demonstrated improved exercise performance, as evidenced by increased running distance twice (*p* = 0.0008) in Tgf‐β1 KO mice. Deletion of CD206^+^ M2 macrophage‐specific Tgf‐β1 stimulates fibro‐adipogenic progenitors (FAPs), inducing Follistatin (*Fst*) expression by 1.70‐fold (*p* = 0.04) and follistatin‐like protein 1 (*Fstl1*) by 2.60‐fold (*p* = 0.01) in tibialis anterior (TA), thus enhancing myogenesis. The Tgf‐β1 KO mice showed increased muscle fibre type I (*Myh7* by 2.40‐fold, *p* = 0.005), muscle fibre type IIa (*Myh2* by 1.50‐fold, *p* = 0.003), type IIx (*Myh1* by 2.35‐fold, *p* = 0.002) in soleus and type II (*Myh4* by 1.76‐fold, *p* = 0.02) in TA. In Tgf‐β1 KO mice, insulin‐stimulated Akt phosphorylation was significantly increased in adipose tissue by 2.14‐fold (*p* = 0.0003) and in the liver by 1.62‐fold (*p* = 0.01). Besides, the Tgf‐β1 KO mice showed increased circulating adiponectin by 1.23 fold (*p* = 0.003), thereby activating the AMPK/SIRT1/PGC‐1α pathway with the phosphorylation level of AMPKα increased by 1.5‐fold (*p* = 0.02), and PGC1α protein level increased by 2.2‐fold (p = 0.02) via increased *AdipoR1* mRNA expression by 1.8‐fold in TA, *p* = 0.0001 and by 1.8‐fold in soleus, *p* = 0.01 in skeletal muscle, leading to improved mitochondrial function in skeletal muscle.

**Conclusions:**

The CD206^+^ M2 macrophage‐specific Tgf‐β1 deletion ameliorates obesity‐induced muscle dysfunction, potentially via two distinct mechanisms. Firstly, it enhances myogenesis by promoting FAP‐mediated expression of *Fst* and *Fstl1*, thereby augmenting myogenesis‐related gene expression in skeletal muscle. Secondly, it also induces adipocytes to produce and secrete adiponectin into the bloodstream, thereby enhancing mitochondrial function via the AMPK/SIRT1/PGC‐1α pathway.

## 1 Introduction

Obesity and Type 2 diabetes are global health issues that affect various tissues and organs, including skeletal muscle, through systemic insulin resistance, lipid metabolism, inflammation and oxidative stress. It is well known that high‐fat diet (HFD)–induced obesity enhances skeletal muscle atrophy, leading to loss of muscle mass and reduced motor function [1, 2, S1]. During obesity, excess fat accumulation leads to the redistribution of fat to the visceral fat and also to other tissues like fatty infiltrations into skeletal muscles, triggering chronic inflammation resulting in decreased overall muscle strength and functionality, termed sarcopenic [3, S2].

It has been reported that the immune system is an important source of cytokines and other secreted factors in the circulation, which can, at least in part, contribute to the regulation of myogenesis [4, 5, S3]. The functions of macrophages in adipose tissue and skeletal muscle may regulate the development of sarcopenic obesity in a closely interconnected manner, even though these conditions are distinct. Obesity impairs immune cells, including macrophages, rendering them dysfunctional and accelerating their detrimental effects, which contribute to multiple metabolic disorders. These dysregulated macrophages also disrupt skeletal muscle metabolism, ultimately leading to muscle dysfunction. The immune system has been reported to play an important role in skeletal muscle growth and regeneration in both acute and chronic injury models [6, 7, 8, S4–S7].

The pro‐inflammatory M1 macrophage phenotype is initially activated in response to injury and secretes chemokines that promote satellite cell proliferation and differentiation. Therefore, inhibiting pro‐inflammatory macrophage activity at an early stage can worsen tissue damage and delay muscle repair [7, 9, S6, S8]. Meanwhile, anti‐inflammatory M2 macrophages secrete factors that are believed to promote satellite cell differentiation and extracellular matrix reconstruction, remodelling and repair [6, 7, 8, S4–S7]. However, persistent pro‐inflammatory activity may impair muscle health; thus, transition to an M2 macrophage phenotype is essential to support tissue growth and restore homeostasis [9, 10, S9]. Notably, in both mice and human studies, anti‐inflammatory macrophages, including CD206‐positive ones, accumulate in ageing muscle tissue and are associated with reducing muscle mass [11, S10–S12]. Mesenchymal stem cells, like progenitors, known as fibro‐adipogenic progenitors (FAPs), reside in muscle cells, which secrete various paracrine factors to support the recovery of muscle following injury [12, 13, S13–S15]. The study on the recovery of muscle injury system demonstrated that depletion of CD206^+^ M2 macrophages stimulated FAPs to express follistatin (Fst), probably via inhibiting activin signalling in the myogenesis process, thus promoting muscle repair via transforming growth factors beta (Tgf‐β) signalling [14]. However, the role of macrophage‐derived Tgf‐β1 in skeletal muscle function during sarcopenic obesity remains unclear.

Besides, mitochondrial dysfunction has been implicated as a potential mediator of sarcopenia [15]. Previous studies demonstrated that the ageing process was associated with a decline in the capacity of the AMPK signalling pathway to respond to various stimuli [16]. AMPK serves as the body's energy sensor [17] and can mediate the expression of SIRT1/PGC‐1α, which is a central regulator of the mitochondrial biogenesis pathway and related metabolism [18]. Therefore, regulating mitochondrial biogenesis could be a potential therapy to improve muscle strength in sarcopenic obesity. Adipose tissue has been reported as a source of regenerative cells that enhance skeletal muscle repair after injury [19]. Adiponectin is a circulating hormone that activates the AMPK/SIRT1/PGC‐1α pathway, thereby inducing mitochondrial biogenesis in the skeletal muscle [20, 21, 22, S16]. Previous studies have shown that depletion of CD206^+^ M2 macrophages improves glucose metabolism and insulin sensitivity in lean mice by increasing *Adipoq* expression in white adipose tissue through inhibition of Tgf‐β signalling [23]. These findings suggest that CD206^+^ M2 macrophages may play an important role in the adipose tissue muscle axis that regulates muscle function. However, the mechanism through which M2 macrophage‐derived Tgf‐β1 contributes to obesity‐related skeletal muscle dysfunction remains unknown. This study aims to elucidate the molecular mechanisms underlying improved skeletal muscle function during obesity‐induced skeletal muscle impairment by deleting the Tgf‐β1 from CD206^+^ M2 macrophages.

## 2 Methods

### 2.1 Mice

CD206CreER^T2^ mice are crossed with Tgf‐β1^flox/flox^ (Tgf‐β1^f/f^) to get CD206CreER^T2^; Tgf‐β1^f/f^ as previously described by Nawaz et al. [14]. All animals were housed in a group of four mice in one cage, under a 12‐h light/dark cycle, 24° ± 2°, humidity (55 ± 5 percentage) automatically controlled. The HFD, 60% Calories Fat (Research Diets, Japan) and water ad libitum were freely available to all mice.

All experiments and procedures were approved by the Animal Care Committee of the University of Toyama (Approved number A2023MED‐16).

### 2.2 Genotyping

Direct PCR (tail) lysis buffer solution (Viagen Biotech) and proteinase K (Roche Diagnostics) were used for tail lysis, following the manufacturer's instructions to obtain whole genomic DNA. A Tks Gflex DNA polymerase kit from TAKARA (Shiga, Japan) was used with this crude DNA to perform PCR.

PCR conditions for CD206CreER^T2^ included Segment 1: 94° for 1 min (one cycle) and Segment 2: 98° for 10 s, 58° for 30 s and 68° for 30 s (30 cycles). Then, PCR products were kept at 4°. The expected DNA fragment size is 299 bp. The primers used for PCR had the sequence GGTCGATGCAACGAGTGATGAG (Primer 1) and GTGAAACAGCATTGCTGTCACTTGG (Primer 2).

The PCR condition for Tgf‐β1^f/f^ included Segment 1: 94° for 1 min (one cycle) and Segment 2: 98° for 10 s, 54° for 30 s and 68° for 30 s (40 cycles). Then, PCR products were kept at 4°. The expected DNA fragment sizes of WT and floxed mice were 210 and 338 bp, respectively. The primers' sequences were AAGACCTGGGTTGGAAGTG (Primer 1) and CTTCTCCGTTTCTCTGTCACCCTAT (Primer 2). Both primers for PCR were purchased from Invitrogen Life Technology (Tokyo, Japan). PCR products were separated using 1.5% agarose gel (Nippon gel) electrophoresis for 35 min. Ethidium bromide (1:1000) was added to visualize DNA on the gel.

### 2.3 Tamoxifen Administration

The tamoxifen (TAM; Sigma‐Aldrich) was dissolved in sunflower oil (WAKO) and incubated at 55° with a vortex every 5 min until completely dissolved. After complete dissolution, TAM was administered as previously described [S17] to both Tgf‐β1^f/f^ and CD206CreER^T2^; Tgf‐β1^f/f^ mice at a dose of 225 mg/kg body weight (BW) for 5 consecutive days at 6 weeks of age, to delete the *Tgfb1* gene (Tgf‐β1 KO).

### 2.4 Glucose Tolerance and Insulin Tolerance Test

Mice were forced to fast for 5 h before doing an intraperitoneal glucose tolerance test (ip‐GTT). Glucose was injected intraperitoneally into both Tgf‐β1 KO and Tgf‐β1^f/f^ at a dose of 1 mg/kg BW.

For the intraperitoneal insulin tolerance test (ip‐ITT), mice were fasted for 4 h. Both Tgf‐β1 KO and Tgf‐β1^f/f^ mice were injected with insulin (Humulin R) with a dose of 1.2 units/kg.

The blood glucose level was measured at 0, 15, 30, 60, 90 and 120 min. In both ip‐GTT and ip‐ITT, the blood glucose level was taken from the tail vein using the STAT STRIP Express 900 (Nova Biomedical, Waltham, MA).

### 2.5 Exercise Tolerance Test

An exercise tolerance test was performed using a procedure previously reported [S18], with slight modifications. In brief, on the first day, the mice were placed on a treadmill with a stimulus device consisting of a shock grid attached to the rear of the belt for 10 min at 10 m/min. The next day, the test was performed after mice had been denied food access for 2 h. The speed and incline are described in Figure S1. The distance run and the number of shocks (intensity of 1 mA) were recorded every 5 min, and a mouse was considered exhausted and removed from the treadmill when it could no longer run and lay down on the shock grid for 15 s. The exhaustion distance was calculated in the following format:

s=v×t

where

s = distance (m).

v = speed (m/min).

t = time (min).

### 2.6 Hanging Time and Grip Strength Test

Hanging time was calculated manually using a net. The mice were trained 1 day before the actual test. On the test day, the mice were fasted for 2 h. The mice were forced to hang on the net till it dropped. The procedure was repeated during the 10‐min test. The maximum hanging time in one drop during 10 min of hanging and the number of drops during 10 min were recorded. A grip strength test was performed by using a small animal grip strength device (Melquest, Toyama, Japan). The mice were trained 1 day before the actual test. The mice were fasted for 2 h before the actual test. The mean value was calculated from three readings per mouse.

### 2.7 Body Composition

The MR VivoLVA Small Animal MRI system (Japan REDOX, Hakata, Fukuoka, Japan) was used as previously described [S19]. The animals were anaesthetized to perform MRI experiments to analyse body fat/lean mass composition. MRI images were analysed using ImageJ 1.53a software to measure the fat/lean mass area.

### 2.8 Western Blotting

Tissues for the western blot analysis were quickly frozen in liquid nitrogen and preserved at −80°C until the analysis. The western blot analysis was performed as described previously [S20]. Briefly, the tissues for western blotting were homogenized in lysis buffer containing 25 mM Tris–HCl (pH 7.4), 10 mM Na_3_VO_4_, 100 mM NaF, 50 mM Na_4_P_2_O_7_, 10 mM EDTA, 0.2% cocktail inhibitor (1 mg/mL) and 2 mM phenylmethylsulfonylfluoride using a Multi‐Beads Shocker cell disrupter (Yasui Kikai Corporation, Osaka, Japan). The lysates were centrifuged to remove any insoluble materials and mixed with loading buffer before protein denaturation by boiling at 95°C for 3 min. The protein content in all the samples was adjusted to a concentration of 2 μg/μL. The protein lysates were run on gels (Mini‐PROTEAN TGX Precast Gels) and transferred to PVDF membrane. Immobilon‐P transfer membranes (Millipore, Billerica MA). The membranes were incubated overnight at 4°C with the primary antibody (1:1000–2000 dilution) and for 2 h at room temperature with the secondary antibody (1:2000 dilution) before being subjected to western blot detection reagent immediately, followed by image development. All antibodies used were added into Table S1. The images were taken by Bio‐Rad ChemiDoc Touch MP.

### 2.9 Enzyme‐Linked Immunosorbent Assay (ELISA) of Adiponectin Level in Serum

Blood samples were collected and centrifuged at 2000 × g for 10 min at 4°. Then, serum was collected and stored at −80° for subsequent assay. The concentration of serum adiponectin was determined by enzyme‐linked immunosorbent assay (ELISA) by using a Mouse Adiponectin ELISA kit (Proteintech), followed by the manufacturer's instructions. Optical density was measured at 450 nm.

### 2.10 Real‐Time Polymerase Chain Reaction (RT‐PCR)

Soleus, gastrocnemius (GC), tibialis anterior (TA) and adipose tissue were collected and extracted using the Qiagen RNeasy kit following the manufacturer's instructions. TaKaRa PrimerScript RNA Kit was used with the company's guidance for reverse transcription. The quantitative PCR amplification reaction was performed using gene‐specific primers (provided in Table S2) and TB Green Fast Premix (Takara, Shiga Japan), followed by the manufacturer's instructions. The relative mRNA expression levels were calculated by ∆∆Ct values were normalized to the internal control Rpl13a or β‐actin.

### 2.11 Skeletal Muscle Single‐Cell Preparation

The single cell of skeletal muscle was isolated and prepared by using a previously reported method with a minor modification [24, 25]. Briefly, tissue was collected and subsequently digested in a mixture of 2 mg/mL collagenase Type II (Gibco) and Dipase (Gibco) for 45 min at 37°C and then filtered through a 100‐μm strainer to harvest single cells.

### 2.12 Magnetic‐Activated Cell Sorting Study

Single cells were dissociated from the skeletal muscle (TA). The single cells were processed for magnetic cell sorting using anti‐PDGFRα microbeads, and the positive fraction was collected. All incubation and procedures were performed at 4° for 10–15 min following the manufacturer's instructions.

### 2.13 Histology

After collection, tissue was fixed in 4% PFA, and paraffin sections were prepared with 5–10 μm thickness and then mounted on the slide. For haematoxylin and eosin (H&E) staining, the slide was stained with H&E. H&E staining from TA and adipose tissue (eWAT) was captured using Keyence BZ‐X800 with a 10× or 20× lens (scale bar 100 μm).

### 2.14 Immunohistochemistry

Paraffin‐embedded and frozen tissue sections were used in immunohistochemical staining. The primary and secondary antibodies were used following the manufacturer's instructions, with the ratio for primary antibody being 1:100, the secondary antibody being 1:250 and DAPI being 1:400. Primary antibodies included CD206 (Santa Cruz Biotechnology) and TGF‐β1 (Santa Cruz Biotechnology). All primer sources were provided in Table S1. All images were taken by an LSM 900 with an Arycan confocal microscope.

### 2.15 Quantification of Adipocyte and Muscle Fibre Size

Quantification of adipocytes and skeletal muscle fibre size was performed in Python using a standardized image analysis pipeline. Skeletal muscle sections were imaged at 20× magnification, and adipose tissue sections at 10× magnification all images were saved as TIFF files. Image processing included pre‐processing, automated cell segmentation, morphometric feature extraction and unit conversion. Cell area in physical units was calculated using the following:

$$\text{Area}\;\left({\mathrm{\mu m}}^{2}\right)=\text{area}\;\left(\text{pixels}\right)\times {\left(\text{pixel size}\right)}^{2}$$

where the pixel size was 0.7576 μm/pixel for 10× adipose tissue and 0.3769 μm/pixel for 20× skeletal muscle images. Four random images were taken for each case, and four animals were used in each case.

### 2.16 Statistical Analysis

Statistical significance between the Tgf‐β1 KO and Tgf‐β1^f/f^ groups was assessed using two‐way ANOVA, followed by the Sidak's multiple‐comparison test for GTT and ITT. Data represent two‐way ANOVA followed by the Holm–Sidak's multiple comparison test for Akt signalling. Data represent one‐way ANOVA followed by Tukey's multiple comparison test where more than two groups were compared. Other data used a two‐tailed unpaired *t*‐test; *p < 0.05, **p < 0.01, ***p < 0.001, ****p < 0.0001. Data are expressed as mean ± SEM.

## 3 Results

### 3.1 Deletion of CD206^+^ M2 Macrophage‐Specific Tgf‐β1 Improves Exercise Performance

**FIGURE 1:**
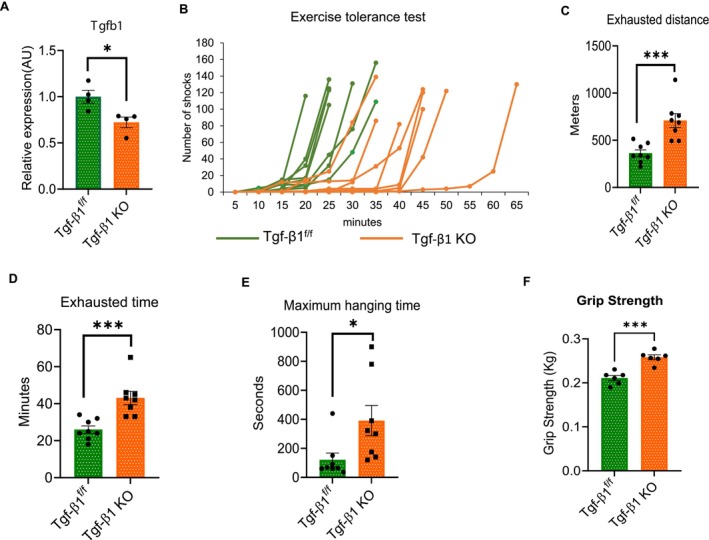
Deletion of CD206^+^ M2 macrophage‐specific Tgf‐β1 improves exercise performance. (A) Relative mRNA expression of the *Tgfb1* gene in TA muscle tissue (*n* = 4, 4). (B) Exercise tolerance test. Individual animal performance is shown, the x‐axis indicates running duration (minutes), and the y‐axis indicates the number of electric shocks recorded at 5‐min intervals (*n* = 8, 8). (C,D) Average running distance and average running time until exhaustion (*n* = 8, 8). (E) Maximum hanging time recorded within a 10‐min period (*n* = 8, 8). (F) Grip strength measured using a small‐animal grip strength device (Melquest, Toyama, Japan). Data are presented as mean ± SEM. Statistical analysis was performed using a two‐tailed unpaired *t*‐test (*p < 0.05, **p < 0.01, ***p < 0.001).

To determine whether M2 macrophage‐derived Tgf‐β1 affects muscle strength, the CD206 M2 macrophages‐specific Tgf‐β1 conditional deletion mouse model was used, both Tgf‐β1^f/f^ and CD206CreER^T2^;Tgf‐β1^f/f^ were treated with TAM at 6 weeks of age. One week later, they were put on an HFD. Exercise tolerance and grip strength tests were performed after 11 weeks of HFD. One week later, glucose and insulin tolerance tests were examined. At the age of 22 weeks, mice were sacrificed and analysed according to the schematic protocol in Figure S2A. Before and after five TAM treatments, the body weights of both groups did not differ significantly (Figure S2B,C). Both groups' body weight and food intake during 12 weeks of HFD were consistently comparable (Figure S2D,E). To evaluate the mouse model, *Tgfb1* gene expression was analysed in TA skeletal muscle and found that it decreased significantly in Tgf‐β1 KO mice (Figure 1A). For further confirmation, immunohistochemistry stained with CD206 and TGF‐β1 data revealed that CD206 and TGF‐β1 double‐positive signals were decreased in TA of Tgf‐β1 KO mice (Figure S2F). Then, an exercise tolerance test was performed until exhaustion using the schematic protocol shown in Figure S1. The result demonstrated that Tgf‐β1 KO mice improve exercise capacity (Figure 1B) with increased exhausted distance and time (Figure 1C,D). Next, grip strength was evaluated by measuring hanging time, as shown in Figure S3A and through a small animal grip strength device (Melquet, Toyama, Japan) as shown in Figure S3B. The maximum hanging time per turn and the number of drops were measured over a 10‐min period. The data showed that Tgf‐β1 KO mice had a longer maximum hanging time with fewer drops over 10 min (Figures 1E and S3C). In addition, grip strength was significantly higher in Tgf‐β1 KO mice (Figure 1F). Collectively, these data showed improved skeletal muscle function in Tgf‐β1 KO mice under HFD‐fed obese conditions.

### 3.2 Deletion of CD206^+^ M2 Macrophage‐Specific Tgf‐β1 Improves Glucose Metabolism

**FIGURE 2:**
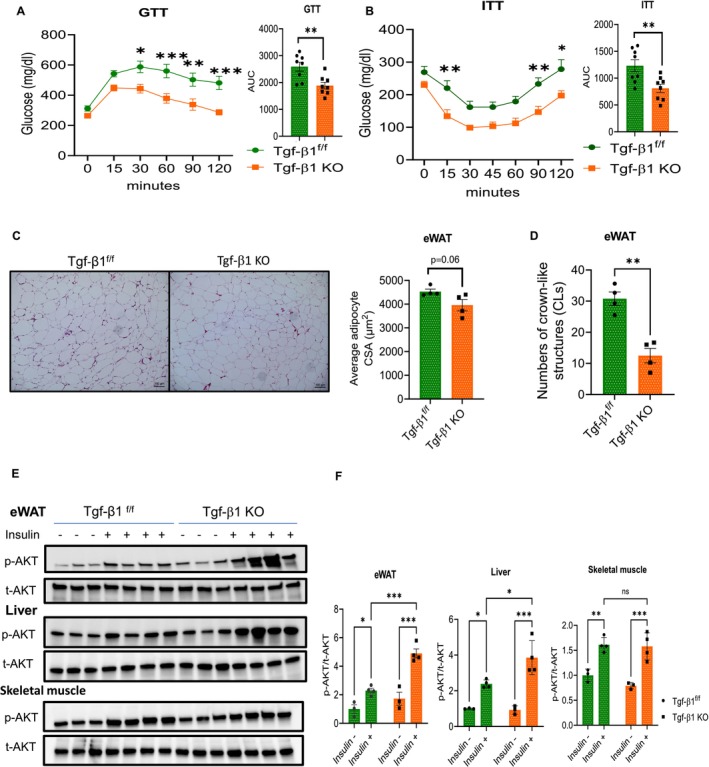
Deletion of CD206^+^ M2 macrophage‐specific Tgf‐β1 improves glucose and insulin tolerance and enhances insulin‐stimulated Akt phosphorylation. (A) Glucose tolerance test (GTT) and corresponding area under the curve (AUC) (*n* = 8, 8). (B) Insulin tolerance test (ITT) and corresponding AUC (*n* = 8, 8). Data represent mean ± SEM. Statistical analysis for GTT and ITT was performed using two‐way ANOVA, followed by Sidak's multiple comparison test (*p < 0.05, **p < 0.01, ***p < 0.001). (C) Representative haematoxylin and eosin (H&E)–stained images of epididymal white adipose tissue (eWAT) from Tgf‐β1^f/f^ and Tgf‐β1 KO obese mice (scale bar, 100 μm; *n* = 4, 4). The right panel shows quantification of average adipocyte size. (D) Quantification of crown‐like structures (CLs) in eWAT from Tgf‐β1^f/f^ and Tgf‐β1 KO mice (*n* = 4, 4). (E) Representative western blot images showing insulin‐stimulated Akt phosphorylation in eWAT, liver and skeletal muscle (gastrocnemius; GC) obtained from Tgf‐β1^f/f^ and Tgf‐β1 KO obese mice (insulin (−): *n* = 3, 3; insulin (+): *n* = 4, 4). (F) Quantification of Western blot signals for eWAT, liver and skeletal muscle. Data represent mean ± SEM. Statistical analysis was performed using two‐way ANOVA followed by Holm–Sidak's multiple comparison test (*p < 0.05, **p < 0.01, ***p < 0.001). Other datasets were analysed using a two‐tailed unpaired *t*‐test (**p < 0.01).

To evaluate obesity, fat mass percentage was evaluated by MRI. The data showed a reduced fat mass ratio in Tgf‐β1 KO obese mice (Figure S4A). Body and tissue weights at the time of sacrifice were also measured, body weight and eWAT were comparable, and iWAT and liver showed significantly less weight in Tgf‐β1 KO obese mice (Figure S4B,C). Next, GTT and ITT were performed, in both tests glucose levels of Tgf‐β1 KO mice were lower, indicating that Tgf‐β1 KO mice had improved glucose and insulin tolerance (Figure 2A,B). Histological data of eWAT showed a slightly higher frequency percentage (%) of smaller adipocytes with reduced tendency of average CSA size of adipocytes (*p* = 0.06) (Figures 2C and S4D) and significant reduction in crown‐like structures (CLs) accumulation (Figures 2D and S4E). In Tgf‐β1 KO obese mice, insulin‐stimulated Akt phosphorylation was significantly enhanced in adipose tissue (eWAT) and liver, whereas in skeletal muscle (GC), it was comparable to control mice (Figure 2E,F). Thus, the deletion of Tgf‐β1 derived from CD206^+^ M2 macrophages improves glucose metabolism and enhances insulin sensitivity.

### 3.3 Deletion of CD206^+^ M2 Macrophage‐Derived Tgf‐β1 Ameliorates Muscle Waste

**FIGURE 3:**
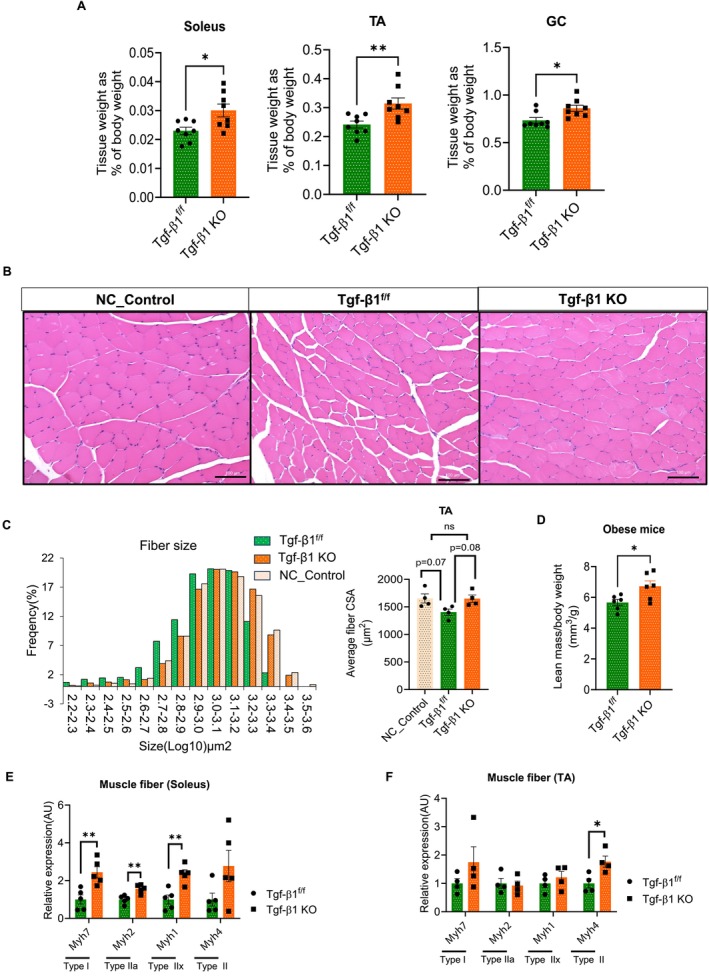
Deletion of CD206^+^ M2 macrophage‐derived Tgf‐β1 ameliorates obesity‐induced muscle wasting. Eight‐week‐old Tgf‐β1f/f and Tgf‐β1 KO mice were fed a high‐fat diet (HFD) for 12 weeks. Mice were then sacrificed, and muscle mass, histology and gene expression were examined. (A) Percentage of tissue weight of soleus, TA and GC muscles at the time of sacrifice (*n* = 8, 8). (B) Representative H&E‐stained images of TA muscle from normal chow control (NC_Control), Tgf‐β1^f/f^ and Tgf‐β1 KO mice (scale bar = 100 μm; *n* = 4, 4, 4). (C) Frequency distribution of muscle fibre cross‐sectional area (CSA, μm^2^) in NC_Control, Tgf‐β1^f/f^ and Tgf‐β1 KO mice. The right panel shows the average CSA (*n* = 4, 4, 4). Statistical analysis was performed using one‐way ANOVA, followed by Tukey's multiple‐comparison test. (D) Lean mass ratio normalized to body weight, measured from one femoral skeletal muscle region using MRI in HFD‐fed obese mice. Muscle area was quantified using ImageJ software (*n* = 6, 6). (E) Relative mRNA expression of muscle fibre–related genes in soleus (*n* = 5, 5). (F) Relative mRNA expression of muscle fibre–related genes in TA (*n* = 4, 4). Data represent mean ± SEM. Statistical analysis was performed using a two‐tailed unpaired *t*‐test (*p < 0.05, **p < 0.01).

Generally, there are two types of muscle fibres. Type I or red muscle (soleus), with a high level of myoglobin and abundant mitochondria, is fatigue‐resistant. Meanwhile, Type II or white muscle (TA), with a low level of myoglobin and fewer mitochondria, is easier to fatigue. Next, the effect of Tgf‐β1 deletion on muscle fibres was investigated. First, the skeletal muscle ratio to body weight was evaluated; the data showed increased skeletal muscle (soleus, TA and GC) percentage to body weight in Tgf‐β1 KO obese mice (Figure 3A). To evaluate obesity‐induced sarcopenia, NC‐fed lean mice (NC_Control) were also examined parallel with Tgf‐β1^f/f^ and Tgf‐β1 KO mice, for muscle fibre cross‐sectional area (CSA) using H&E staining. Analysis of CSA frequency distribution and average fibre size revealed a higher proportion of smaller muscle fibres in Tgf‐β1^f/f^ obese mice compared to NC_Control and Tgf‐β1 KO mice. Whereas, the mean CSA tended to be reduced in Tgf‐β1^f/f^ (*p* = 0.07) and preserved in Tgf‐β1 KO mice (*p* = 0.99) compared with NC_Control (Figure 3C). In addition, the lean mass ratio was also measured by MRI of leg skeletal muscle; the data showed that there was a significantly higher lean mass ratio in Tgf‐β1 KO mice in the obese state, whereas no difference was observed in the lean state, compared to Tgf‐β1^f/f^ mice (Figures 3D and S5). Consistently, both muscle fibre type–related genes were increased significantly in the soleus muscle of Tgf‐β1 KO mice (Figure 3E). Additionally, gene expression analysis revealed that muscle fibre type II gene *Myh4* was markedly increased in the TA of Tgf‐β1 KO mice (Figure 3F). These data suggest that deletion of Tgf‐β1 might protect from muscle waste induced by diet‐induced obesity. Additionally, gene expression analysis of extracellular matrix and collagen‐related genes revealed that Tgf‐β1 KO mice significantly reduce fibrosis (Figure S6). Thus, deletion of the CD206^+^ M2 macrophage‐derived Tgf‐β1 is critical for regulating obesity‐induced skeletal muscle loss.

### 3.4 Deletion of the Tgf‐β1 Gene From CD206^+^ M2 Macrophage Stimulates FAPs to Express Fst, Thus Enhancing Myogenesis in Skeletal Muscle

**FIGURE 4:**
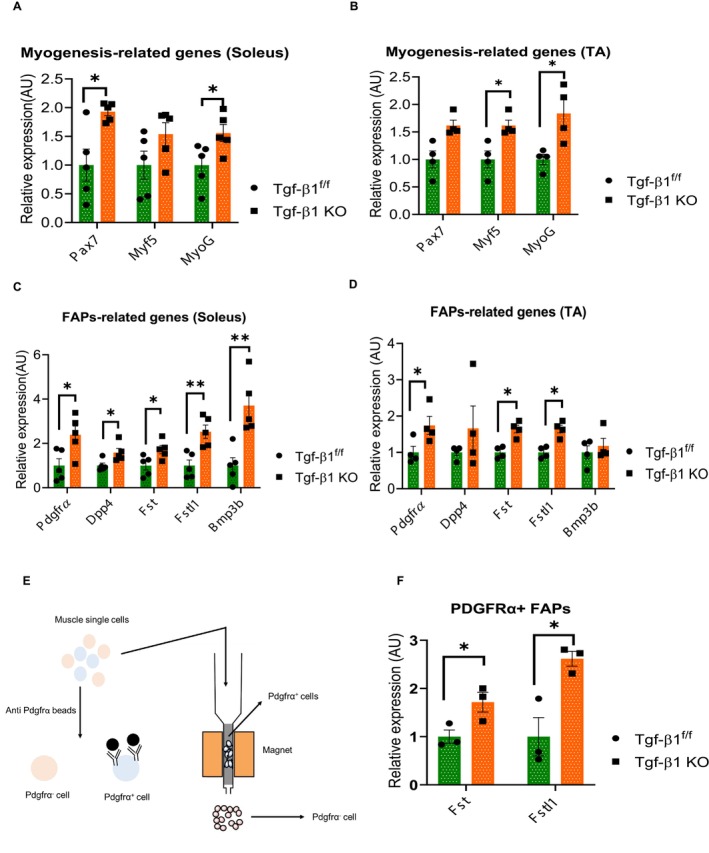
Deletion of CD206^+^ M2 macrophages‐specific Tgf‐β1 stimulates FAPs to express *Fst*, thereby enhancing myogenesis in skeletal muscle. (A) Relative mRNA expression of myogenesis‐related genes in soleus (*n* = 5, 5). (B) Relative mRNA expression of myogenesis‐related genes in TA (*n* = 4, 4). (C) Relative mRNA expression of fibro‐adipogenic progenitors (FAPs)–related genes in soleus (*n* = 5, 5). (D) Relative mRNA expression of FAPs‐related genes in TA (*n* = 4, 4). (E) Schematic protocol for magnetic‐activated cell sorting (MACS). Single cells were dissociated from TA muscle, incubated with anti‐platelet‐derived growth factor alpha (anti‐PDGFRα) microbeads and subjected to MACS to isolate the PDGFRα‐positive population. (F) Relative mRNA expression of FAPs‐related genes in the PDGFRα‐positive FAPs (*n* = 3, 3). Data represent mean ± SEM. Statistical analysis was performed using a two‐tailed unpaired *t*‐test (*p < 0.05, **p < 0.01).

To determine how deletion of Tgf‐β1 derived from CD206^+^ M2 macrophages increases muscle mass, the myogenesis‐related genes were investigated in soleus and TA. The data showed that myogenesis‐related genes in soleus and TA, were upregulated significantly in Tgf‐β1 KO mice (Figure 4A,B), because deletion of CD206^+^ M2 macrophages‐specific Tgf‐β1 was reported to activate FAPs, which then secrete the factors that enhance myogenesis after injury [14]. Next, the FAPs‐related gene expressions were evaluated in both soleus and TA and found that they were increased considerably in both soleus and TA of Tgf‐β1 KO mice, especially *Fst* and folistatin‐related protein 1 (*Fstl1*), suggesting that deletion of Tgf‐β1 from CD206^+^ M2 macrophages stimulated FAPs to express *Fst* and *Fstl1*, thus improving myogenesis (Figure 4C,D). For further confirmation, FAPs were purified using magnetically activated cell sorting (MACS) to purify platelet‐derived growth factor receptor alpha (PDGFRα)–positive populations, a marker of FAPs [24, 25, S21, S22], from TA, as shown in Figure 4E. Consistently, *Fst* and *Fstl1* expression levels were found to be increased in PDGFRα^+^ FAPs isolate from Tgf‐β1 KO mice (Figure 4F), supporting the notion that Tgf‐β1 suppresses *Fst* gene expression through inhibition of FAPs activation. It is reported that Fst can induce muscle hypertrophy by promoting satellite cell proliferation by inhibition of either myostatin or activin [26]. Collectively, these data showed that the deletion of the CD206^+^ M2 macrophage‐specific Tgf‐β1 stimulated FAPs to secrete Fst, thereby enhancing myogenesis in skeletal muscle during obesity‐induced muscle loss.

### 3.5 Deletion of CD206^+^ M2 Macrophage‐Specific Tgf‐β1 Gene Stimulated Mitochondrial Biogenesis and FA Oxidation in Skeletal Muscle

**FIGURE 5:**
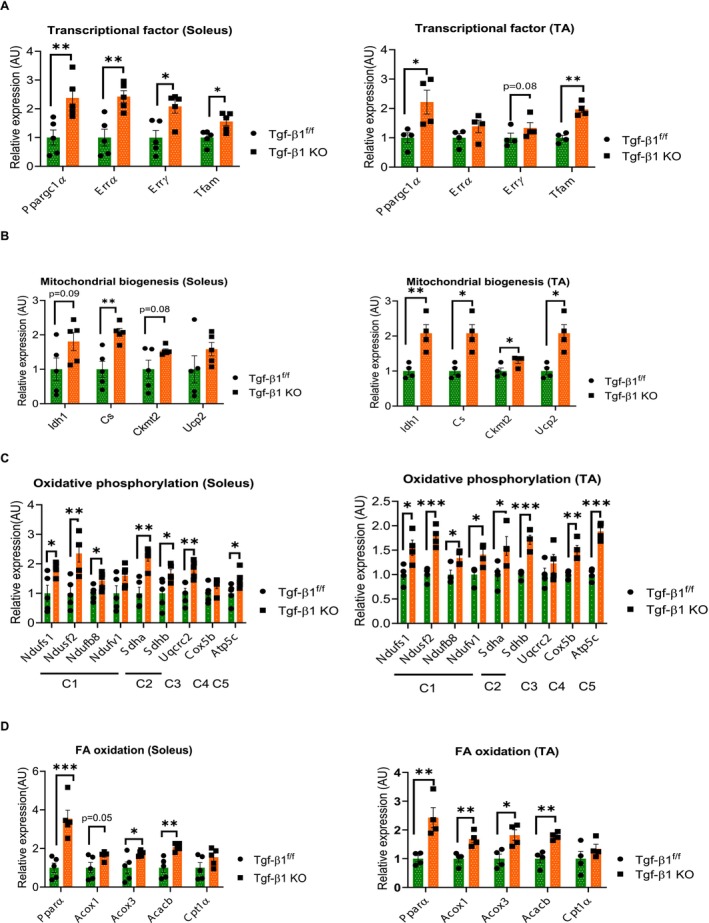
Deletion of CD206^+^ M2 macrophage‐specific Tgf‐β1 gene stimulated mitochondrial biogenesis and FA oxidation in skeletal muscle. (A) Relative mRNA expression of mitochondrial transcription factor–related genes in soleus (left panel; *n* = 5, 5) and TA (right panel; *n* = 4, 4). (B) Relative mRNA expression of mitochondrial biogenesis–related genes in soleus (left panel; *n* = 5, 5) and TA (right panel; *n* = 4, 4). (C) Relative mRNA expression of oxidative phosphorylation–related genes in soleus (left panel; *n* = 5, 5) and TA (right panel; *n* = 4, 4). (D) Relative mRNA expression of fatty acid (FA) oxidation‐related genes in soleus (left panel; *n* = 5, 5) and TA (right panel; *n* = 4, 4). Data represent mean ± SEM. Statistical analysis was performed using a two‐tailed unpaired *t*‐test (*p < 0.05, **p < 0.01, ***p < 0.001).

Sarcopenia is associated with muscle loss and reduced mitochondrial function [15]. Thus, mitochondrial function in skeletal muscle is more critical for regulation of various functions in skeletal muscle including soleus, TA and GC. Skeletal muscles obtained from Tgf‐β1 KO mice exhibited elevated expression of transcriptional and mitochondrial biogenesis‐related genes (Figure 5A,B and S7A,B). Next, the gene expression analysis of oxidative phosphorylation–related genes from five complexes was conducted to examine how they coordinate mitochondrial function at the transcriptional level. The data showed that almost all complexes were activated in Tgf‐β1 KO mice (Figures 5C and S7C). The fatty acid (FA) oxidation–related gene expression analysis revealed that these genes were also significantly upregulated in Tgf‐β1 KO mice (Figures 5D and S7D). In addition, FA uptake‐related gene expressions were also upregulated in soleus muscle obtained from Tgf‐β1 KO mice (Figure S8). Thus, deletion of Tgf‐β1 in CD206^+^ M2 macrophages enhances mitochondrial function and promotes oxidative metabolism by upregulation of FA oxidation and uptake.

### 3.6 Deletion of CD206^+^ M2 Macrophage‐Specific Tgf‐β1 Activates AMPK/SIRT1/PGC‐1α Pathway in Skeletal Muscle

**FIGURE 6:**
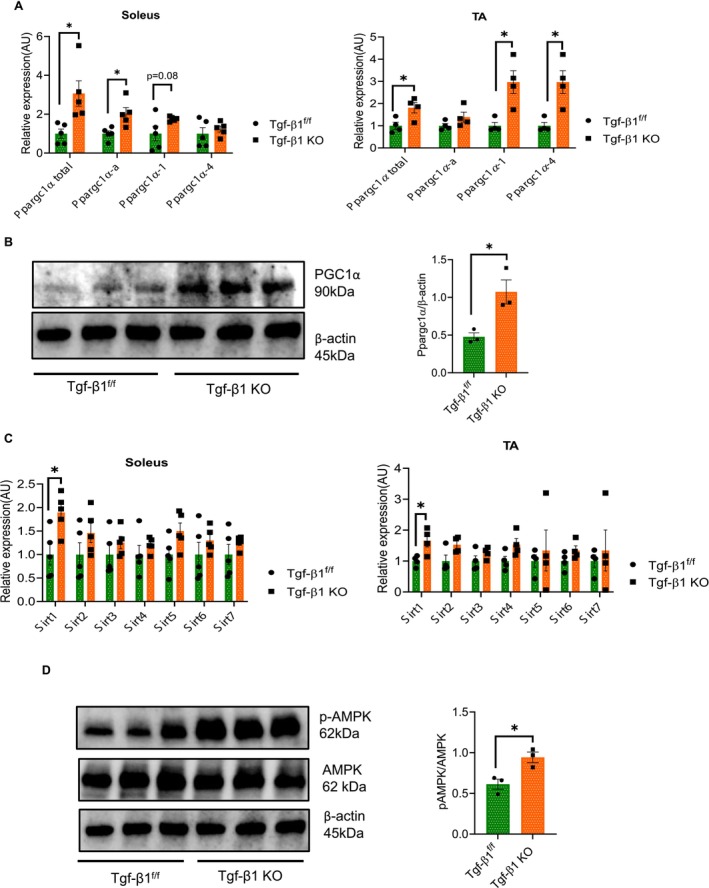
Deletion of CD206^+^ M2 macrophage‐specific Tgf‐β1 activates the AMPK/SIRT1/PGC‐1α pathway in skeletal muscle. (A) Relative mRNA expression of *Ppargc1α* isoforms in soleus (left panel; *n* = 5, 5) and TA (right panel; *n* = 4, 4). (B) Western blot analysis of PGC1α protein levels in skeletal muscle (left panel) and quantification of PGC1α protein normalized to β‐actin (right panel; *n* = 3, 3). (C) Relative mRNA expression of sirtuin family‐related genes in soleus (left panel; *n* = 5, 5) and TA (right panel; *n* = 4, 4). (D) Western blot analysis of AMPKα phosphorylation (Thr172) in skeletal muscle (TA) (left panel) and quantification of p‐AMPKα normalized to total AMPK (right panel; *n* = 3, 3). Data represent mean ± SEM. Statistical analysis was performed using a two‐tailed unpaired *t*‐test (*p < 0.05).

To investigate how deletion of CD206^+^ M2 macrophage‐specific Tgf‐β1 improved mitochondrial function, the peroxisome proliferator–activated receptor γ coactivator‐1α (*Ppargc1α*/PGC1α) expression was examined, which is a transcriptional coactivator and a central inducer of mitochondrial biogenesis in cells [27]. As expected, *Ppargc1α* gene expression was increased in Tgf‐β1 KO mice in soleus, TA, and GC tissue (Figures 6A and S9A). In addition, a protein level of PGC1α revealed by western blotting was also increased in Tgf‐β1 KO mice (Figure 6B). Next, Sirt‐related gene expression was evaluated, as sirtuins have been proposed to act as a potential activator of PGC1α transcriptional activity [28]. Predictably, Sirt1 gene expression was also significantly increased in Tgf‐β1 KO mice (Figures 6C and S9B). The SIRT1/PGC‐1α were activated through the AMPK pathway. Consistently, the data of phosphorylation level of AMPKα subnit Th172 was increased in Tgf‐β1 KO mice (Figures 6D and S9C). Because AMPK was reported to regulate glucose levels and promote glucose transporter 4 (*Glut4*) gene *Slc2a4* [18, S23], the data showed a significant upregulation of the expression of *Slc2a4* in skeletal muscle (Figure S9D–F). Collectively, these data showed that deletion of CD206^+^ M2 macrophages specific Tgf‐β1 activated the AMPK/SIRT1/PGC‐1α pathway.

### 3.7 Deletion of CD206^+^ M2 Macrophage‐Derived Tgf‐β1 Gene Stimulated Adipose Tissue to Secrete Circulating Adiponectin That Controls Homeostasis

**FIGURE 7:**
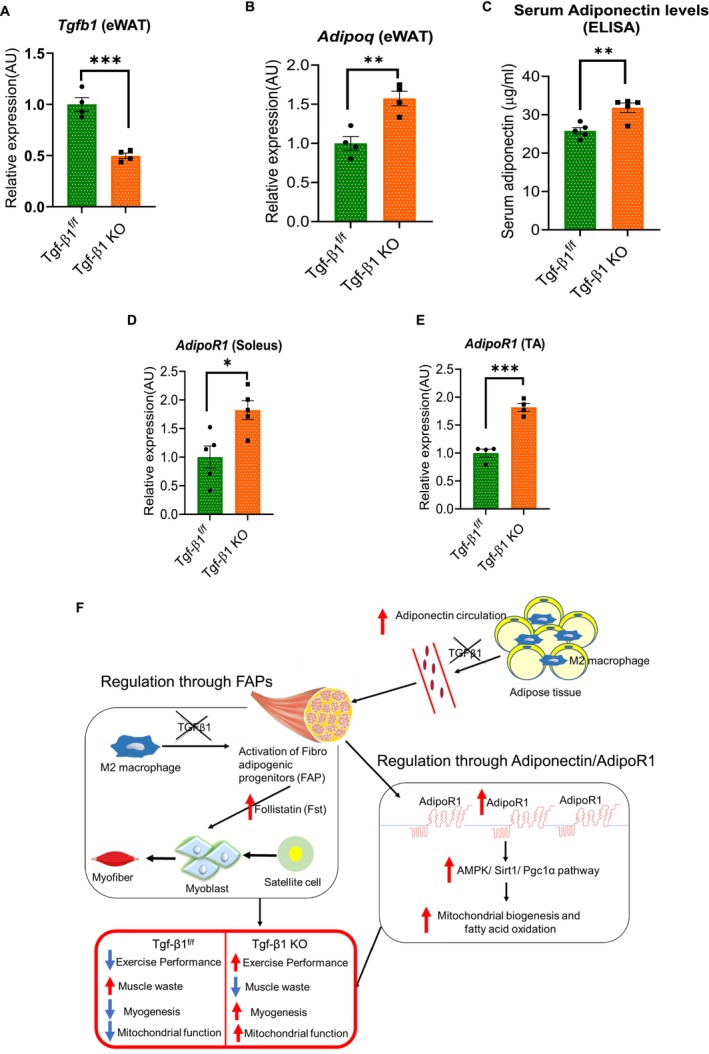
Deletion of CD206^+^ M2 macrophage‐derived Tgf‐β1 stimulates adipose tissue to secrete circulating adiponectin. (A) Relative mRNA expression of *Tgfb1*in eWAT (*n* = 4, 4). (B) Relative mRNA expression of *Adipoq* in eWAT (*n* = 4, 4). (C) Quantification of serum adiponectin levels measured by ELISA (*n* = 5, 5). (D) Relative mRNA expression of *AdipoR1* in soleus (*n* = 5, 5). (E) Relative mRNA expression of *AdipoR1* in TA (*n* = 4, 4). (F) Graphical abstract summarizing the proposed mechanism. Data represent mean ± SEM. Statistical analysis was performed using a two‐tailed unpaired *t*‐test (*p < 0.05, **p < 0.01, ***p < 0.001).

A recent study showed that partial depletion of CD206^+^ M2 macrophages upregulated metabolically favourable genes, especially adiponectin within adipose tissue, via inhibition of TGF‐β signalling in the NC‐fed lean mice [23]. Other studies also demonstrated that circulating adiponectin can activate AMPK signalling in skeletal muscle by inducing *AdipoR1* expression [21, 22]. To know the mechanism of how mitochondrial biogenesis and the AMPK/SIRT1/PGC‐1α pathway are enhanced, adiponectin was evaluated, which might be involved in activating AMPK in an AdipoR‐dependent manner. Thus, the first *Tgfb1* gene expression level was evaluated in epididymal white adipose tissue (eWAT), and it was found to be downregulated considerably in Tgf‐β1 KO mice (Figure 7A). Adiponectin (*Adipoq*) gene expression in eWAT was also increased in Tgf‐β1 KO mice (Figure 7B). The circulating adiponectin level was significantly enhanced in Tgf‐β1 KO mice (Figure 7C). Because adiponectin can stimulate AdipoR1 in skeletal muscle, *AdipoR1* expression in skeletal muscle (soleus, TA and GC) was elevated significantly in Tgf‐β1 KO mice (Figures 7D,E and S9G). Thus, the deletion of the CD206^+^ M2 macrophage‐specific Tgf‐β1 gene stimulates adipose tissue to secrete adiponectin and enhances mitochondrial function in skeletal muscle via the activated AMPK pathway via *AdipoR1*.

## 4 Discussion

Obesity and sarcopenia are closely interrelated; during obesity, there is a molecular‐level metabolic adaptation, suppression of insulin signalling, increased adipose tissue inflammation, redistribution of fat and fatty infiltration into muscle, leading to reduced muscular renewal capacity and progression to sarcopenia [29]. Macrophages are critical for muscle recovery from injury, and macrophage‐derived signals regulate the proliferation of muscle stem cells [14]. Several reports showed that the role of interleukin 6 (IL6), a pro‐inflammatory factor secreted by M1 macrophages, negatively correlated with muscle mass and muscle strength in sarcopenia [30, 31, S4, S6, S24, S25]. Tumour necrosis factor‐alpha (TNFα), another M1 macrophage‐derived factor, has been reported to contribute to muscle wasting and weakness by accelerating protein degradation in mature muscle [32]. Meanwhile, it was reported that depletion of CD206^+^ M2 macrophages stimulates FAPs to express *Fst*, thereby promoting muscle recovery after injury [14], suggesting that CD206^+^ M2 macrophages might be involved in myogenesis during sarcopenic obesity. Patient living with Type 2 diabetes were reported to reduce the proportion of muscle fibre type I and increase the proportion of muscle fibre type II [33]. *Fst* has been reported to enhance skeletal muscle growth by inhibiting the myostatin and activin A signalling pathways, whereas deletion of FAP‐derived Fst results in reduced exercise capacity and impaired locomotor function [S26]. Interestingly, current data showed that deletion of Tgf‐β1 in CD206^+^ M2 macrophages increased muscle fibre type I in the soleus and improved systemic glucose metabolism and insulin sensitivity. Additionally, this study also demonstrated that deletion of CD206^+^ M2 macrophage‐derived Tgf‐β1 ameliorates muscle waste due to enhanced myogenesis by stimulating FAPs to express *Fst*, resulting in increased exercise capacity (Figure 7F).

Besides, mitochondrial dysfunction has been identified as a hallmark of aging [34]. AMPK, PGC‐1α and SIRT1 were considered prominent regulators of mitochondrial function. AMPK has an essential role in mitochondrial biogenesis [35, S27], and its biological activity is reduced during sarcopenia [16]. SIRT1, a potential activator of PGC‐1α transcriptional activity, can directly activate PGC1α in the cytoplasm through lysine deacetylation [36, S28, S29]. Both of them activated the co‐activator mitochondrial transcription factor A (Tfam) [37, S30], which directly binds to target mtDNA and activates the replication and transcription of the corresponding regions, resulting in elevated expression of mitochondrial biogenesis‐related genes. It is reported that the expression of *PGC1α, ERRα* and other related factors was reduced in sarcopenia in human [38]. Thus, it is reasonable to propose that AMPK, PGC‐1α and SIRT1 pathways coordinately regulate mitochondrial biogenesis, thus ameliorating sarcopenic obesity. The current study provided evidence that the AMPK/SIRT1/PGC‐1α pathway is activated in the muscles of Tgf‐β1 KO mice.

Metabolism of the whole body was controlled by the interaction between organs, tissue and cell types, especially adipose tissue, muscle and liver, through cytokines or direct interorgan communication [19, 39]. Meanwhile, adipose tissue is the predominant site of fat storage and engages in interorgan crosstalk to coordinate the cellular response to extrinsic lipid signals [40, S31]. The current study showed improved insulin‐stimulated Akt phosphorylation in eWAT and liver with higher expression of *Adipoq* in eWAT of Tgfβ1 KO mice. Previous studies demonstrated that adiponectin in blood circulation can activate the AMPK pathway by activating AdipoR1 signals in skeletal muscle [21, 22, 40]. Consistently, the data showed that deletion of the CD206^+^ M2 macrophage‐specific Tgf‐β1 gene stimulated adipose tissue to express and secrete adiponectin, increasing circulating adiponectin concentration, thereby activating the AdipoR1, AMPK/SIRT1/PGC‐1α pathway and improving mitochondrial function in skeletal muscle.

## 5 Conclusions

In conclusion, obesity‐related skeletal muscle dysfunction was improved in Tgf‐β1 KO mice, possibly through two independent mechanisms (Figure 7F). First, deletion of CD206^+^ M2 macrophage‐derived Tgf‐β1 stimulated FAPs to express *Fst* and *Fstl1*, thereby promoting myogenesis. Second, it enhanced adipocyte expression and secretion of adiponectin into the circulation, thereby supporting improved muscle function. This study also has limitations, as it does not clarify the relative contribution of each mechanism to the overall improvement in muscle strength. Further studies will be required to dissect the extent to which each pathway drives.

## Funding

This study was supported by Moonshot R&D (JPMJMS2021), Japan Society for the Promotion of Science (24K02502, 22K203737 and 24K19282, 21K16338 and 23KJ1022, 22K16423 and 24K19303 and 22K16424), Uehara Memorial Foundation 2023, Eli Lilly Japan KK Innovation Research Grant, Suzuken Memorial Foundation, Japan Diabetes Society, Japan Foundation for Applied Enzymology, Naito Foundation, Japan Diabetes Foundation, Japan Society for the Study of Obesity, First Bank of Toyama Scholarship Foundation, Yamaguchi Endocrine Research Foundation, Japan Association for Diabetes Education and Care, Boehringer Ingelheim, Novo Nordisk Pharma, Lotte Foundation, Hokugin Young Researchers Grant and ONO Medical Research Foundation.

## Conflicts of Interest

The authors declare no conflicts of interest.

## Supporting information


**Figure S1:** Schematic protocol for exercise test.
**Figure S2:** Experiment protocol, body weight and food intake. (A) Schematic protocol. (B) Body weight before TAM (*n* = 8, 8). (C) Body weight after five doses of TAM (*n* = 8, 8). (D) Body weight during 12 weeks of HFD feeding (*n* = 8, 8). (E) Food intake during 12 weeks of HFD feeding (*n* = 8, 8). (F) Representative confocal images indicate TGF‐β1 co‐localization with CD206 (scale bar = 20 μm, *n* = 4, 4). Arrows indicate CD206/TGF‐β1 double‐positive signals. (G) Quantification of TGF‐β1 + CD206+/total CD206+ (*n* = 4, 4). Data represent mean ± SEM. Statistical analysis was performed using a two‐tailed unpaired *t*‐test (*p < 0.05).
**Figure S3:** Measurement of grip strength. (A) Measurement of hanging time. (B) Measurement of grip strength. (C) Numbers of drops during 10 min of hanging. Data represent mean ± SEM. Statistical analysis was performed using a two‐tailed unpaired *t*‐test (**p < 0.01).
**Figure S4:** (A) Fat mass ratio measured by MRI of HFD‐fed obese mice. (B) Body weight. (C) Tissue weight (grams) eWAT, iWAT and liver (*n* = 8, 8). (D) Adipocyte (eWAT) size cross‐sectional area (μm^2^) distribution frequency (*n* = 4, 4). (E) Representative confocal images indicate F4/80 (green)co‐localization with CD11c (red) (scale bar = 20 μm, *n* = 4, 4). Data represent mean ± SEM. Statistical analysis was performed using a two‐tailed unpaired *t*‐test (*p < 0.05, **p < 0.01).
**Figure S5:**. Lean mass ratio of NC‐fed control mice measured by MRI, normalized by body weight (*n* = 6, 6). Data represent mean ± SEM. Statistical analysis was performed using a two‐tailed unpaired *t*‐test.
**Figure S6:** Fibrosis‐related gene expressions in TA muscle (*n* = 4, 4). Data represent mean ± SEM. Statistical analysis was performed using a two‐tailed unpaired *t*‐test (*p < 0.05, **p < 0.01).
**Figure S7:** Deletion of CD206^+^ M2 macrophage‐specific Tgf‐β1 stimulates mitochondrial biogenesis and FA oxidation in GC. (A) Relative mRNA expression of mitochondrial transcriptional factor–related genes in GC (*n* = 4, 4). (B) Relative mRNA expression of mitochondrial biogenesis–related genes in GC (*n* = 4, 4). (C) Relative mRNA expression of oxidative phosphorylation–related genes in GC (*n* = 4, 4). (D) Relative mRNA expression of FA oxidation–related genes in GC (*n* = 4, 4). Data represent mean ± SEM. Statistical analysis was performed using a two‐tailed unpaired *t*‐test (*p < 0.05, **p < 0.01, ***p < 0.001).
**Figure S8:** FA uptake‐related gene expression in muscle. (A) Relative mRNA expression of FA uptake‐related genes in soleus (*n* = 5, 5). (B) Relative mRNA expression of FA uptake‐related genes in TA (*n* = 4, 4). (C) Relative mRNA expression of FA uptake‐related genes in GC (*n* = 4, 4). Data represent mean ± SEM. Statistical analysis was performed using a two‐tailed unpaired *t*‐test (*p < 0.05, **p < 0.01).
**Figure S9:** Deletion of CD206^+^ M2 macrophage‐specific Tgf‐β1‐activated *Ppargc1α*, *Slc2a4* (*Glut4*) expression in skeletal muscle. (A) Relative mRNA expression of PGC1α‐isoform genes in GC (*n* = 4, 4). (B) Relative mRNA expression of sirtuin‐related genes in GC (*n* = 4, 4). (C) Relative mRNA expression of AMPKα‐related genes in GC (*n* = 4, 4). (D) Relative mRNA expression of *Slc2a4* (*Glut4*) gene in soleus (*n* = 5, 5). (E) Relative mRNA expression of *Slc2a4* (*Glut4*) gene in TA (*n* = 4, 4). (F) Relative mRNA expression of *Slc2a4* (*Glut4*) gene in GC (*n* = 4, 4). (G) Relative mRNA expression of *AdipoR1* gene in GC (*n* = 4, 4). Data represent mean ± SEM. Statistical analysis was performed using a two‐tailed unpaired *t*‐test (*p < 0.05, **p < 0.01, ***p < 0.001).

**Table S1:** Key resources table.
**Table S2:** Primer list.

## References

[1] C. L. Axelrod , W. S. Dantas , and J. P. Kirwan , “Sarcopenic Obesity: Emerging Mechanisms and Therapeutic Potential,” Metabolism 146 (2023): 155639. 37380015 10.1016/j.metabol.2023.155639 PMC11448314

[2] T. Ji , Y. Li , and L. Ma , “Sarcopenic Obesity: An Emerging Public Health Problem,” Aging and Disease 13, no. 2 (2022): 379–388. 35371597 10.14336/AD.2021.1006 PMC8947824

[3] L. Bianchi and S. Volpato , “Muscle Dysfunction in Type 2 Diabetes: A Major Threat to Patient's Mobility and Independence,” Acta Diabetologica 53, no. 6 (2016): 879–889. 27393005 10.1007/s00592-016-0880-y

[4] O. Zamir , P. O. Hasselgren , T. Higashiguchi , J. A. Frederick , and J. E. Fischer , “Tumour Necrosis Factor (TNF) and Interleukin‐1 (IL‐1) Induce Muscle Proteolysis Through Different Mechanisms,” Mediators of Inflammation 1, no. 4 (1992): 247–250. 18475468 10.1155/S0962935192000371 PMC2365344

[5] J. G. Tidball , “Regulation of Muscle Growth and Regeneration by the Immune System,” Nature Reviews. Immunology 17, no. 3 (2017): 165–178. 10.1038/nri.2016.150 PMC5452982 28163303

[6] L. Giordani , G. J. He , E. Negroni , et al., “High‐Dimensional Single‐Cell Cartography Reveals Novel Skeletal Muscle‐Resident Cell Populations,” Molecular Cell 74, no. 3 (2019): 609–621.e6. 30922843 10.1016/j.molcel.2019.02.026

[7] J. G. Tidball and M. Wehling‐Henricks , “Shifts in Macrophage Cytokine Production Drive Muscle Fibrosis,” Nature Medicine 21, no. 7 (2015): 665–666. 10.1038/nm.3896 26151325

[8] S. Willenborg , D. E. Sanin , A. Jais , et al., “Mitochondrial Metabolism Coordinates Stage‐Specific Repair Processes in Macrophages During Wound Healing,” Cell Metabolism 33, no. 12 (2021): 2398–2414.e9. 34715039 10.1016/j.cmet.2021.10.004

[9] L. Arnold , A. Henry , F.¸. Poron , et al., “Inflammatory Monocytes Recruited After Skeletal Muscle Injury Switch Into Antiinflammatory Macrophages to Support Myogenesis,” Journal of Experimental Medicine 204, no. 5 (2007): 1057–1069. 17485518 10.1084/jem.20070075 PMC2118577

[10] D. Ruffell , F. Mourkioti , A. Gambardella , et al., “A CREB‐C/EBPbeta Cascade Induces M2 Macrophage‐Specific Gene Expression and Promotes Muscle Injury Repair,” Proceedings of the National Academy of Sciences of the United States of America 106, no. 41 (2009): 17475–17480. 19805133 10.1073/pnas.0908641106 PMC2762675

[11] Y. Wang , M. Wehling‐Henricks , G. Samengo , and J. G. Tidball , “Increases of M2a Macrophages and Fibrosis in Aging Muscle Are Influenced by Bone Marrow Aging and Negatively Regulated by Muscle‐Derived Nitric Oxide,” Aging Cell 14, no. 4 (2015): 678–688. 26009878 10.1111/acel.12350 PMC4531081

[12] H. Soliman , M. Theret , W. Scott , et al., “Multipotent Stromal Cells: One Name, Multiple Identities,” Cell Stem Cell 28, no. 10 (2021): 1690–1707. 34624231 10.1016/j.stem.2021.09.001

[13] U. Lendahl , L. Muhl , and C. Betsholtz , “Identification, Discrimination and Heterogeneity of Fibroblasts,” Nature Communications 13, no. 1 (2022): 3409. 10.1038/s41467-022-30633-9 PMC9192344 35701396

[14] A. Nawaz , M. Bilal , S. Fujisaka , et al., “Depletion of CD206(+) M2‐Like Macrophages Induces Fibro‐Adipogenic Progenitors Activation and Muscle Regeneration,” Nature Communications 13, no. 1 (2022): 7058. 10.1038/s41467-022-34191-y PMC9678897 36411280

[15] C. López‐Otín , M. A. Blasco , L. Partridge , M. Serrano , and G. Kroemer , “The Hallmarks of Aging,” Cell 153, no. 6 (2013): 1194–1217. 23746838 10.1016/j.cell.2013.05.039 PMC3836174

[16] R. M. Reznick , H. Zong , J. Li , et al., “Aging‐Associated Reductions in AMP‐Activated Protein Kinase Activity and Mitochondrial Biogenesis,” Cell Metabolism 5, no. 2 (2007): 151–156. 17276357 10.1016/j.cmet.2007.01.008 PMC1885964

[17] X. Fu , M. Zhu , S. Zhang , M. Foretz , B. Viollet , and M. Du , “Obesity Impairs Skeletal Muscle Regeneration Through Inhibition of AMPK,” Diabetes 65, no. 1 (2016): 188–200. 26384382 10.2337/db15-0647 PMC4686944

[18] S. Jäger , C. Handschin , J. St.‐Pierre , and B. M. Spiegelman , “AMP‐Activated Protein Kinase (AMPK) Action in Skeletal Muscle via Direct Phosphorylation of PGC‐1alpha,” Proceedings of the National Academy of Sciences of the United States of America 104, no. 29 (2007): 12017–12022. 17609368 10.1073/pnas.0705070104 PMC1924552

[19] Q. Sastourné‐Arrey , M. Mathieu , X. Contreras , et al., “Adipose Tissue Is a Source of Regenerative Cells That Augment the Repair of Skeletal Muscle After Injury,” Nature Communications 14, no. 1 (2023): 80. 10.1038/s41467-022-35524-7 PMC9816314 36604419

[20] Z. V. Wang and P. E. Scherer , “Adiponectin, the Past Two Decades,” Journal of Molecular Cell Biology 8, no. 2 (2016): 93–100. 26993047 10.1093/jmcb/mjw011 PMC4816148

[21] T. Yamauchi , J. Kamon , Y. Minokoshi , et al., “Adiponectin Stimulates Glucose Utilization and Fatty‐Acid Oxidation by Activating AMP‐Activated Protein Kinase,” Nature Medicine 8, no. 11 (2002): 1288–1295. 10.1038/nm788 12368907

[22] M. Iwabu , T. Yamauchi , M. Okada‐Iwabu , et al., “Adiponectin and AdipoR1 Regulate PGC‐1alpha and Mitochondria by Ca(2+) and AMPK/SIRT1,” Nature 464, no. 7293 (2010): 1313–1319. 20357764 10.1038/nature08991

[23] A. Nawaz , A. Aminuddin , T. Kado , et al., “CD206(+) M2‐Like Macrophages Regulate Systemic Glucose Metabolism by Inhibiting Proliferation of Adipocyte Progenitors,” Nature Communications 8, no. 1 (2017): 286. 10.1038/s41467-017-00231-1 PMC5561263 28819169

[24] A. W. Joe , L. Yi , A. Natarajan , et al., “Muscle Injury Activates Resident Fibro/Adipogenic Progenitors That Facilitate Myogenesis,” Nature Cell Biology 12, no. 2 (2010): 153–163. 20081841 10.1038/ncb2015 PMC4580288

[25] A. Uezumi , S. I. Fukada , N. Yamamoto , S. Takeda , and K. Tsuchida , “Mesenchymal Progenitors Distinct From Satellite Cells Contribute to Ectopic Fat Cell Formation in Skeletal Muscle,” Nature Cell Biology 12, no. 2 (2010): 143–152. 20081842 10.1038/ncb2014

[26] H. Gilson , O. Schakman , S. Kalista , P. Lause , K. Tsuchida , and J. P. Thissen , “Follistatin Induces Muscle Hypertrophy Through Satellite Cell Proliferation and Inhibition of Both Myostatin and Activin,” American Journal of Physiology. Endocrinology and Metabolism 297, no. 1 (2009): E157–E164. 19435857 10.1152/ajpendo.00193.2009

[27] S. Austin and J. St‐Pierre , “PGC1α and Mitochondrial Metabolism–Emerging Concepts and Relevance in Ageing and Neurodegenerative Disorders,” Journal of Cell Science 125, no. Pt 21 (2012): 4963–4971. 23277535 10.1242/jcs.113662

[28] B. J. Gurd , “Deacetylation of PGC‐1α by SIRT1: Importance for Skeletal Muscle Function and Exercise‐Induced Mitochondrial Biogenesis,” Applied Physiology, Nutrition, and Metabolism 36, no. 5 (2011): 589–597. 10.1139/h11-070 21888529

[29] C. W. Li , K. Yu , N. Shyh‐Chang , et al., “Pathogenesis of Sarcopenia and the Relationship With Fat Mass: Descriptive Review,” Journal of Cachexia, Sarcopenia and Muscle 13, no. 2 (2022): 781–794. 35106971 10.1002/jcsm.12901 PMC8977978

[30] M. Visser , M. Pahor , D. R. Taaffe , et al., “Relationship of Interleukin‐6 and Tumor Necrosis Factor‐Alpha With Muscle Mass and Muscle Strength in Elderly Men and Women: The Health ABC Study,” Journals of Gerontology. Series A, Biological Sciences and Medical Sciences 57, no. 5 (2002): M326–M332. 11983728 10.1093/gerona/57.5.m326

[31] Y. Xiang , J. Dai , L. Xu , X. Li , J. Jiang , and J. Xu , “Research Progress in Immune Microenvironment Regulation of Muscle Atrophy Induced by Peripheral Nerve Injury,” Life Sciences 287 (2021): 120117. 34740577 10.1016/j.lfs.2021.120117

[32] M. B. Reid and Y. P. Li , “Tumor Necrosis Factor‐Alpha and Muscle Wasting: A Cellular Perspective,” Respiratory Research 2, no. 5 (2001): 269–272. 11686894 10.1186/rr67 PMC59514

[33] A. Oberbach , Y. Bossenz , S. Lehmann , et al., “Altered Fibre Distribution and Fibre‐Specific Glycolytic and Oxidative Enzyme Activity in Skeletal Muscle of Patients With Type 2 Diabetes,” Diabetes Care 29, no. 4 (2006): 895–900. 16567834 10.2337/diacare.29.04.06.dc05-1854

[34] D. Liu , Y. B. Fan , X. H. Tao , et al., “Mitochondrial Quality Control in Sarcopenia: Updated Overview of Mechanisms and Interventions,” Aging and Disease 12, no. 8 (2021): 2016–2030. 34881083 10.14336/AD.2021.0427 PMC8612607

[35] R. Bergeron , J. M. Ren , K. S. Cadman , et al., “Chronic Activation of AMP Kinase Results in NRF‐1 Activation and Mitochondrial Biogenesis,” American Journal of Physiology. Endocrinology and Metabolism 281, no. 6 (2001): E1340–E1346. 11701451 10.1152/ajpendo.2001.281.6.E1340

[36] K. Aquilano , P. Vigilanza , S. Baldelli , B. Pagliei , G. Rotilio , and M. R. Ciriolo , “Peroxisome Proliferator‐Activated Receptor Gamma Co‐Activator 1alpha (PGC‐1alpha) and Sirtuin 1 (SIRT1) Reside in Mitochondria: Possible Direct Function in Mitochondrial Biogenesis,” Journal of Biological Chemistry 285, no. 28 (2010): 21590–21599. 20448046 10.1074/jbc.M109.070169 PMC2898414

[37] A. P. Rebelo , L. M. Dillon , and C. T. Moraes , “Mitochondrial DNA Transcription Regulation and Nucleoid Organization,” Journal of Inherited Metabolic Disease 34, no. 4 (2011): 941–951. 21541724 10.1007/s10545-011-9330-8

[38] E. Migliavacca , S. K. H. Tay , H. P. Patel , et al., “Mitochondrial Oxidative Capacity and NAD(+) Biosynthesis Are Reduced in Human Sarcopenia Across Ethnicities,” Nature Communications 10, no. 1 (2019): 5808. 10.1038/s41467-019-13694-1 PMC6925228 31862890

[39] M. Mogi , K. Kohara , H. Nakaoka , et al., “Diabetic Mice Exhibited a Peculiar Alteration in Body Composition With Exaggerated Ectopic Fat Deposition After Muscle Injury due to Anomalous Cell Differentiation,” Journal of Cachexia, Sarcopenia and Muscle 7, no. 2 (2016): 213–224. 27493874 10.1002/jcsm.12044 PMC4864245

[40] J. H. Stern , J. M. Rutkowski , and P. E. Scherer , “Adiponectin, Leptin, and Fatty Acids in the Maintenance of Metabolic Homeostasis Through Adipose Tissue Crosstalk,” Cell Metabolism 23, no. 5 (2016): 770–784. 27166942 10.1016/j.cmet.2016.04.011 PMC4864949

