# Supplementary material for: Deletion of Tgf‐β1 From CD206+ M2 Macrophages Ameliorates Obesity‐Induced Suppression of Myogenesis and AMPK Phosphorylation in Skeletal Muscle
Source: J Cachexia Sarcopenia Muscle. 2026 Jun 18;17(3):e70322. doi: 10.1002/jcsm.70322 (PMC13280218; doi:10.1002/jcsm.70322)
Supplement: Supplementary file 1 — Figure S1: Schematic protocol for exercise test. Figure S2: Experiment protocol, body weight and food intake. (A) Schematic protocol. (B) Body weight before TAM (n = 8, 8). (C) Body weight after five doses of TAM (n = 8, 8). (D) Body weight during 12 weeks of HFD feeding (n = 8, 8). (E) Food intake during 12 weeks of HFD feeding (n = 8, 8). (F) Representative confocal images indicate TGF‐β1 co‐localization with CD206 (scale bar = 20 μm, n = 4, 4). Arrows indicate CD206/TGF‐β1 double‐positive signals. (G) Quantification of TGF‐β1 + CD206+/total CD206+ (n = 4, 4). Data represent mean ± SEM. Statistical analysis was performed using a two‐tailed unpaired t‐test (*p < 0.05). Figure S3: Measurement of grip strength. (A) Measurement of hanging time. (B) Measurement of grip strength. (C) Numbers of drops during 10 min of hanging. Data represent mean ± SEM. Statistical analysis was performed using a two‐tailed unpaired t‐test (**p < 0.01). Figure S4: (A) Fat mass ratio measured by MRI of HFD‐fed obese mice. (B) Body weight. (C) Tissue weight (grams) eWAT, iWAT and liver (n = 8, 8). (D) Adipocyte (eWAT) size cross‐sectional area (μm2) distribution frequency (n = 4, 4). (E) Representative confocal images indicate F4/80 (green)co‐localization with CD11c (red) (scale bar = 20 μm, n = 4, 4). Data represent mean ± SEM. Statistical analysis was performed using a two‐tailed unpaired t‐test (*p < 0.05, **p < 0.01). Figure S5:. Lean mass ratio of NC‐fed control mice measured by MRI, normalized by body weight (n = 6, 6). Data represent mean ± SEM. Statistical analysis was performed using a two‐tailed unpaired t‐test. Figure S6: Fibrosis‐related gene expressions in TA muscle (n = 4, 4). Data represent mean ± SEM. Statistical analysis was performed using a two‐tailed unpaired t‐test (*p < 0.05, **p < 0.01). Figure S7: Deletion of CD206+ M2 macrophage‐specific Tgf‐β1 stimulates mitochondrial biogenesis and FA oxidation in GC. (A) Relative mRNA expression of mitochondrial transcriptional f [file JCSM-17-e70322-s001.pdf]

Figure S1: Schematic protocol for exercise test.

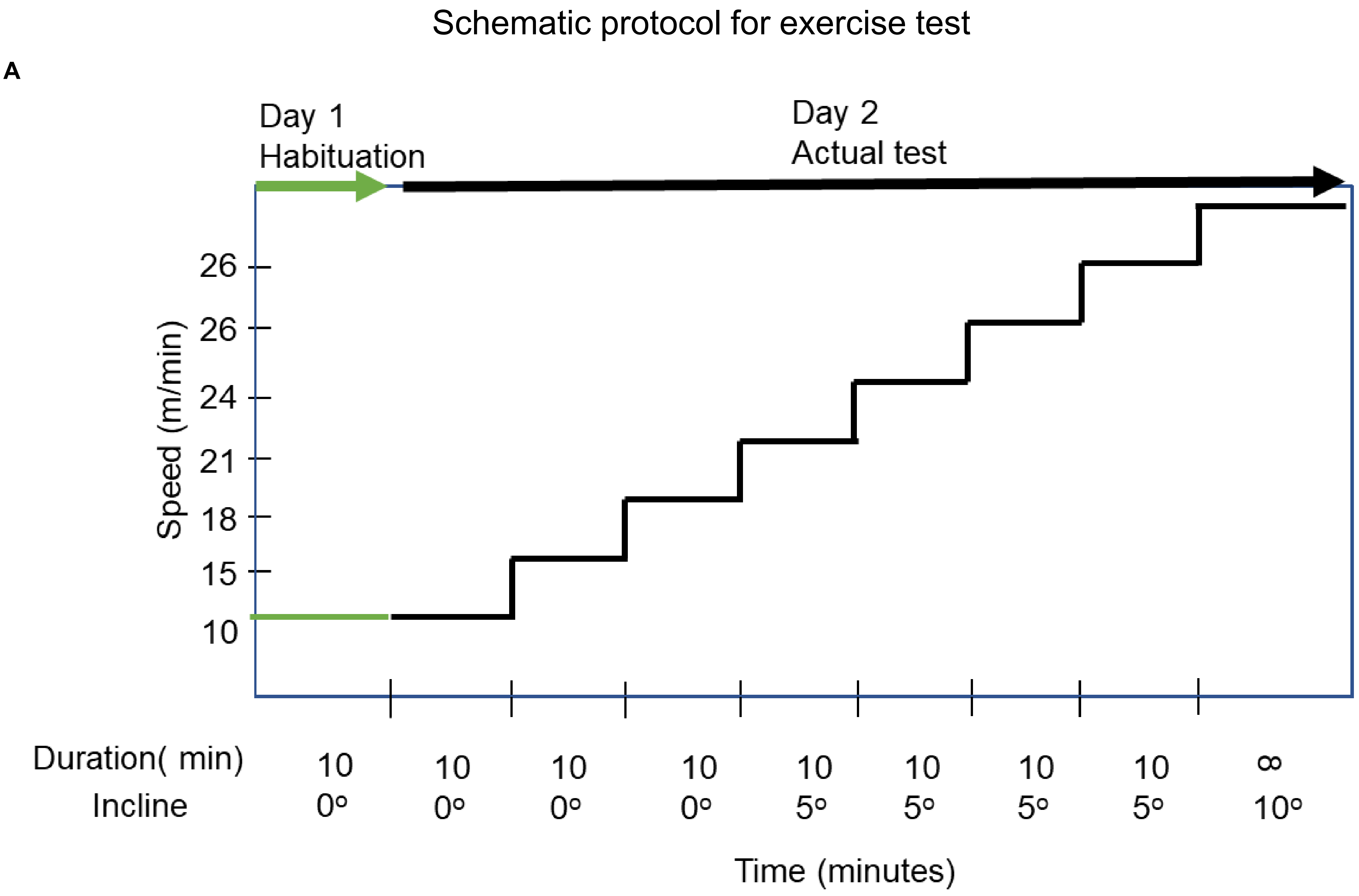

An exercise tolerance test was performed using a procedure reported previously with slight modification [S19]. In brief, after 11 weeks of HFD, mice were examined exercise tolerance test. On the first day, the mice were trained with a treadmill with a stimulus device consisting of a shock grid attached to the rear end of the belt for 10 minutes at the speed of 10 m/min. The next day, the test was performed after mice had been denied access to food for 2h. The speed and incline are described in the above schematic figure. The distance run and the number of shocks obtained over 5-minute intervals were recorded, and a mouse was considered exhausted and removed from the treadmill when it could not run anymore and lay down on the shock grid for 15 seconds. The number of shocks was recorded every 5 minutes. The exhaustion distance was calculated in the below format:

$s = v \times t$

s: distance (m)

v: speed (m/min)

t: time (minutes)

Figure S2

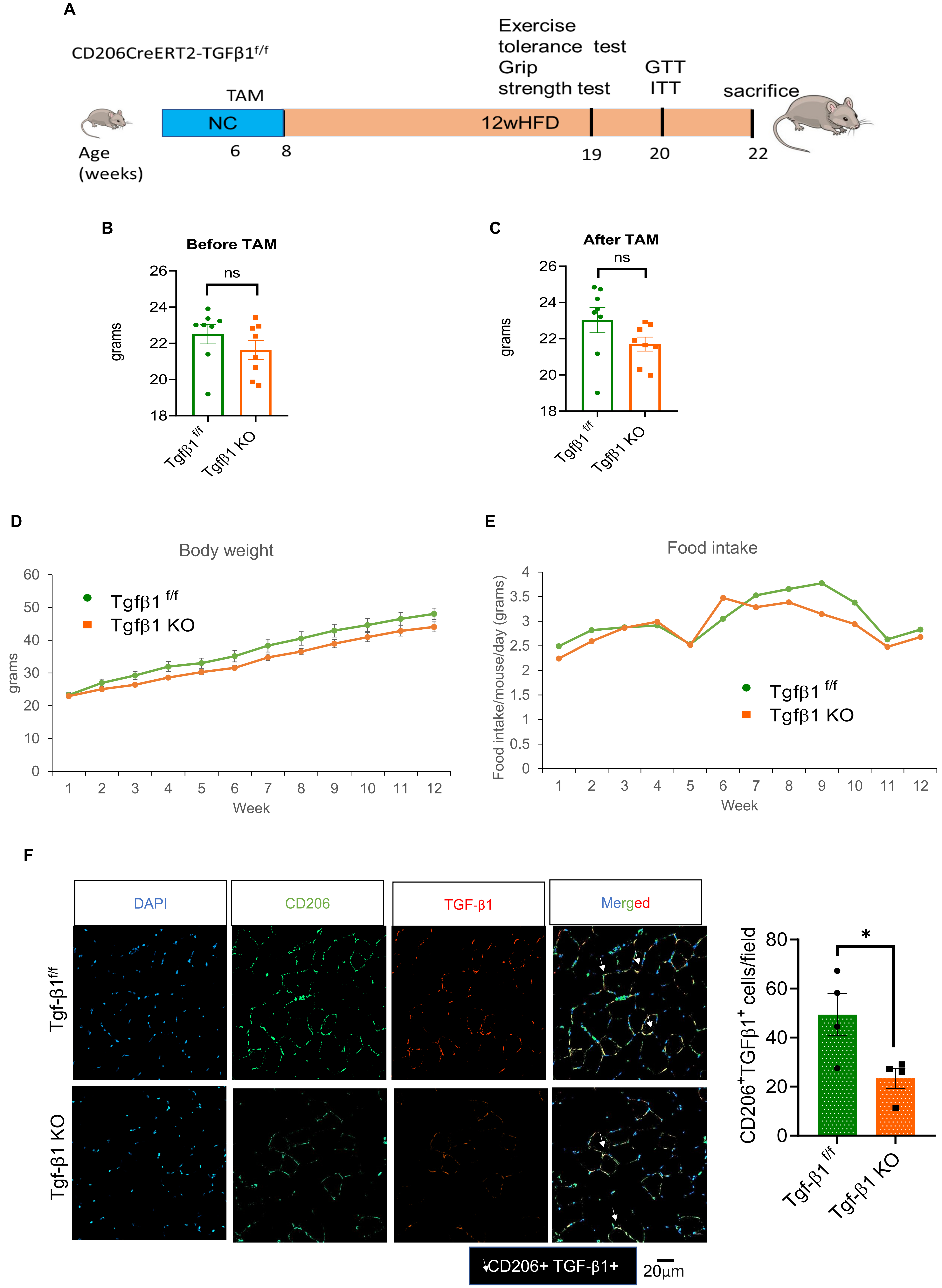
A
CD206CreERT2-TGFβ1f/f
TAM
NC
12wHFD
Exercise tolerance test
Grip strength test
GTT
ITT
sacrifice
Age (weeks)
6
8
19
20
22
B
Before TAM
ns
grams
Tgfβ1 f/f
Tgfβ1 KO
C
After TAM
ns
grams
Tgfβ1 f/f
Tgfβ1 KO
D
Body weight
Tgfβ1 f/f
Tgfβ1 KO
grams
Week
E
Food intake
Food intake/mouse/day (grams)
Tgfβ1 f/f
Tgfβ1 KO
Week
F
DAPI
CD206
TGF-β1
Merged
Tgf-β1f/f
Tgf-β1 KO
CD206+TGFβ1+ cells/field
*
Tgf-β1 f/f
Tgf-β1 KO
CD206+ TGF-β1+
20µm

Figure S2: Experiment protocol, body weight and food intake. (A) Schematic protocol. (B) Body weight before TAM ($n = 8, 8$). (C) Body weight after five doses of TAM ($n = 8, 8$). (D) Body weight during 12 weeks of HFD feeding ($n = 8, 8$). (E) Food intake during 12 weeks of HFD feeding ($n = 8, 8$). (F) Representative confocal images indicate TGF-β1 co-localization with CD206 (scale bar = 20 μm, $n = 4, 4$). Arrows indicate CD206/TGF-β1 double-positive signals. (G) Quantification of TGF-β1 + CD206+/total CD206+ ($n = 4, 4$). Data represent mean ± SEM. Statistical analysis was performed using a two-tailed unpaired *t*-test ($*p < 0.05$).

Figure S3

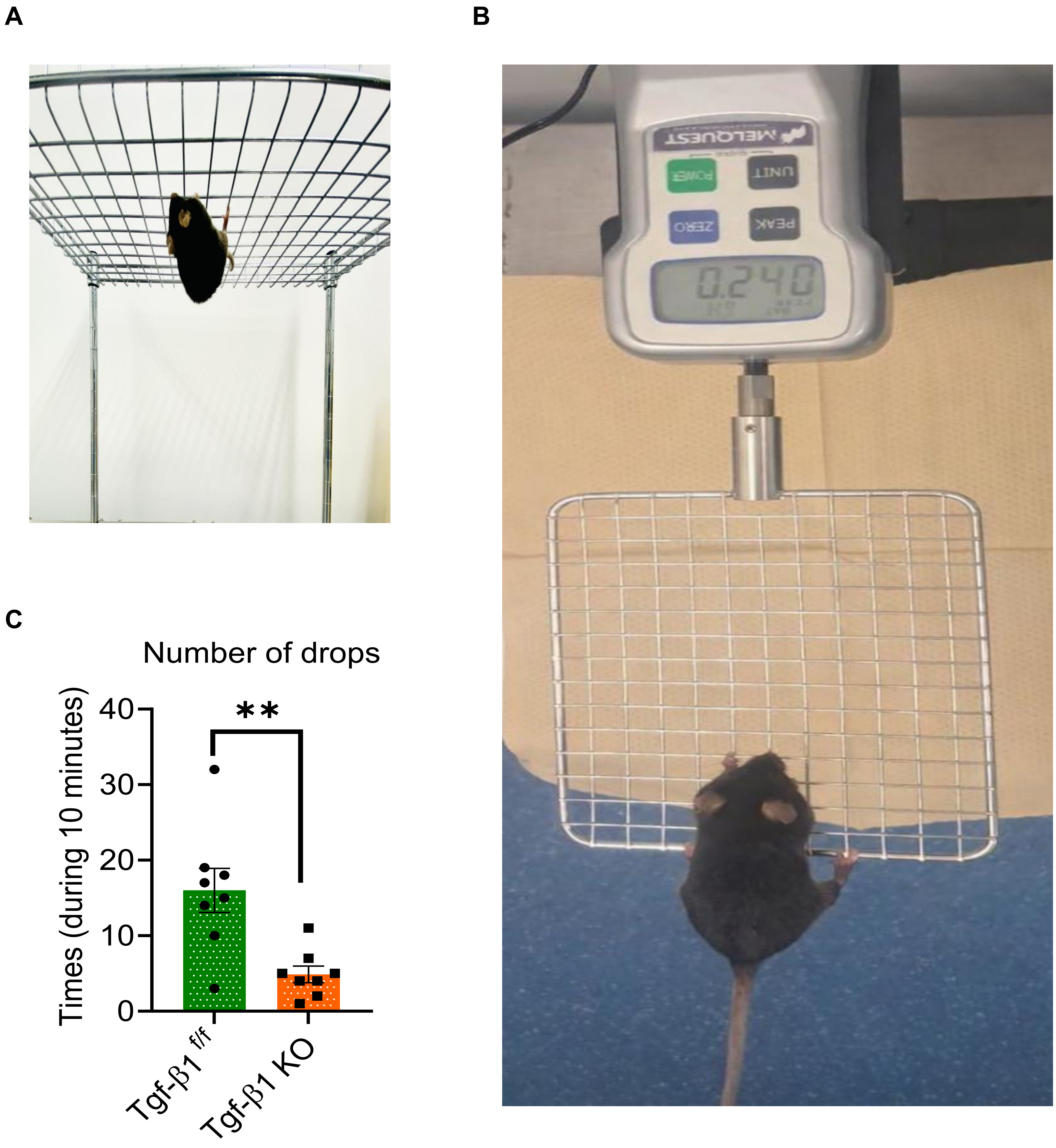

Figure S3: Measurement of grip strength. (A) Measurement of hanging time. (B) Measurement of grip strength. (C) Numbers of drops during 10 min of hanging. Data represent mean ± SEM. Statistical analysis was performed using a two-tailed unpaired *t*-test (** $p < 0.01$).

Figure S4

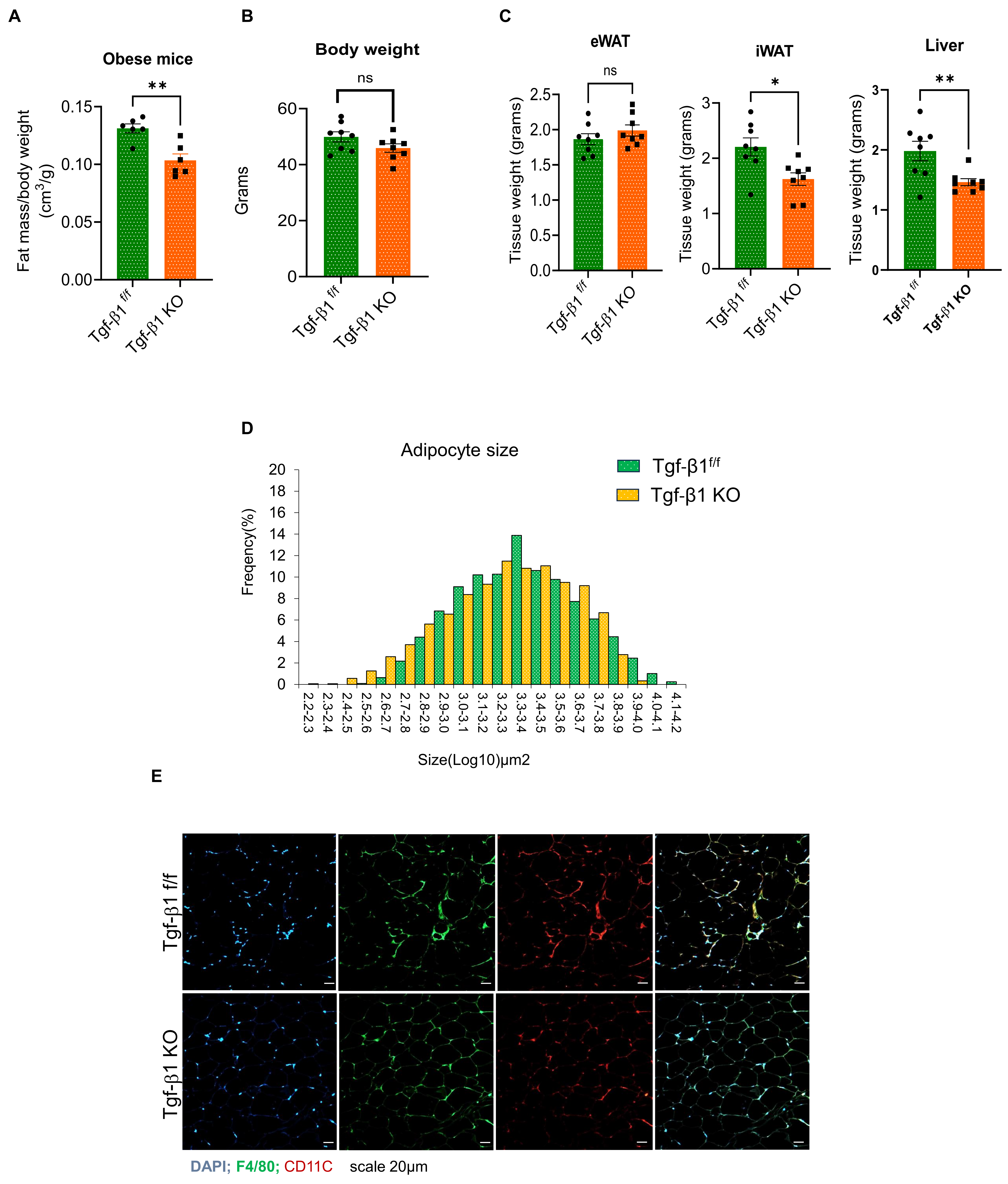

A
Obese mice
Fat mass/body weight (cm3/g)
0.15
0.10
0.05
0.00
**
Tgf-β1 f/f
Tgf-β1 KO
B
Body weight
Grams
60
40
20
0
ns
Tgf-β1 f/f
Tgf-β1 KO
C
eWAT
ns
Tissue weight (grams)
2.5
2.0
1.5
1.0
0.5
0.0
Tgf-β1 f/f
Tgf-β1 KO
iWAT
*
Tissue weight (grams)
3
2
1
0
Tgf-β1 f/f
Tgf-β1 KO
Liver
**
Tissue weight (grams)
3
2
1
0
Tgf-β1 f/f
Tgf-β1 KO
D
Adipocyte size
Tgf-β1f/f
Tgf-β1 KO
Freqency(%)
20
18
16
14
12
10
8
6
4
2
0
2.2-2.3
2.3-2.4
2.4-2.5
2.5-2.6
2.6-2.7
2.7-2.8
2.8-2.9
2.9-3.0
3.0-3.1
3.1-3.2
3.2-3.3
3.3-3.4
3.4-3.5
3.5-3.6
3.6-3.7
3.7-3.8
3.8-3.9
3.9-4.0
4.0-4.1
4.1-4.2
Size(Log10)μm2
E
Tgf-β1 f/f
Tgf-β1 KO
DAPI; F4/80; CD11C scale 20μm

Figure S4: (A) Fat mass ratio measured by MRI of HFD-fed obese mice. (B) Body weight. (C) Tissue weight (grams) eWAT, iWAT and liver (*n* = 8, 8). (D) Adipocyte (eWAT) size cross-sectional area (μm^2) distribution frequency (*n* = 4, 4). (E) Representative confocal images indicate F4/80 (green)co-localization with CD11c (red) (scale bar = 20 μm, *n* = 4, 4). Data represent mean ± SEM. Statistical analysis was performed using a two-tailed unpaired *t*-test ($*p < 0.05$, $**p < 0.01$).

Figure S5

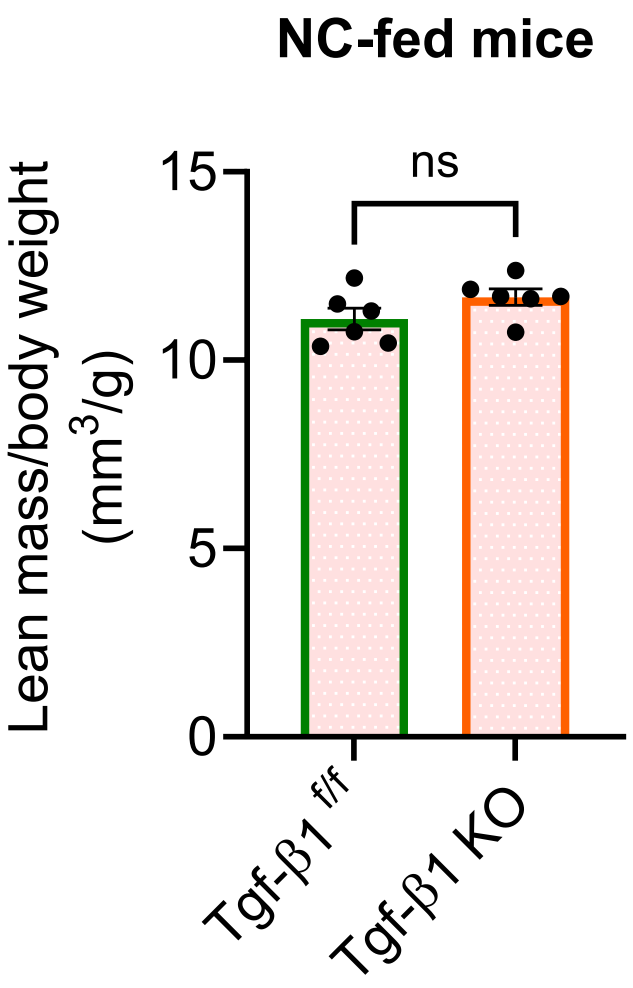

Figure S5:. Lean mass ratio of NC-fed control mice measured by MRI, normalized by body weight ($n = 6$, 6). Data represent mean ± SEM. Statistical analysis was performed using a two-tailed unpaired *t*-test.

Figure S6

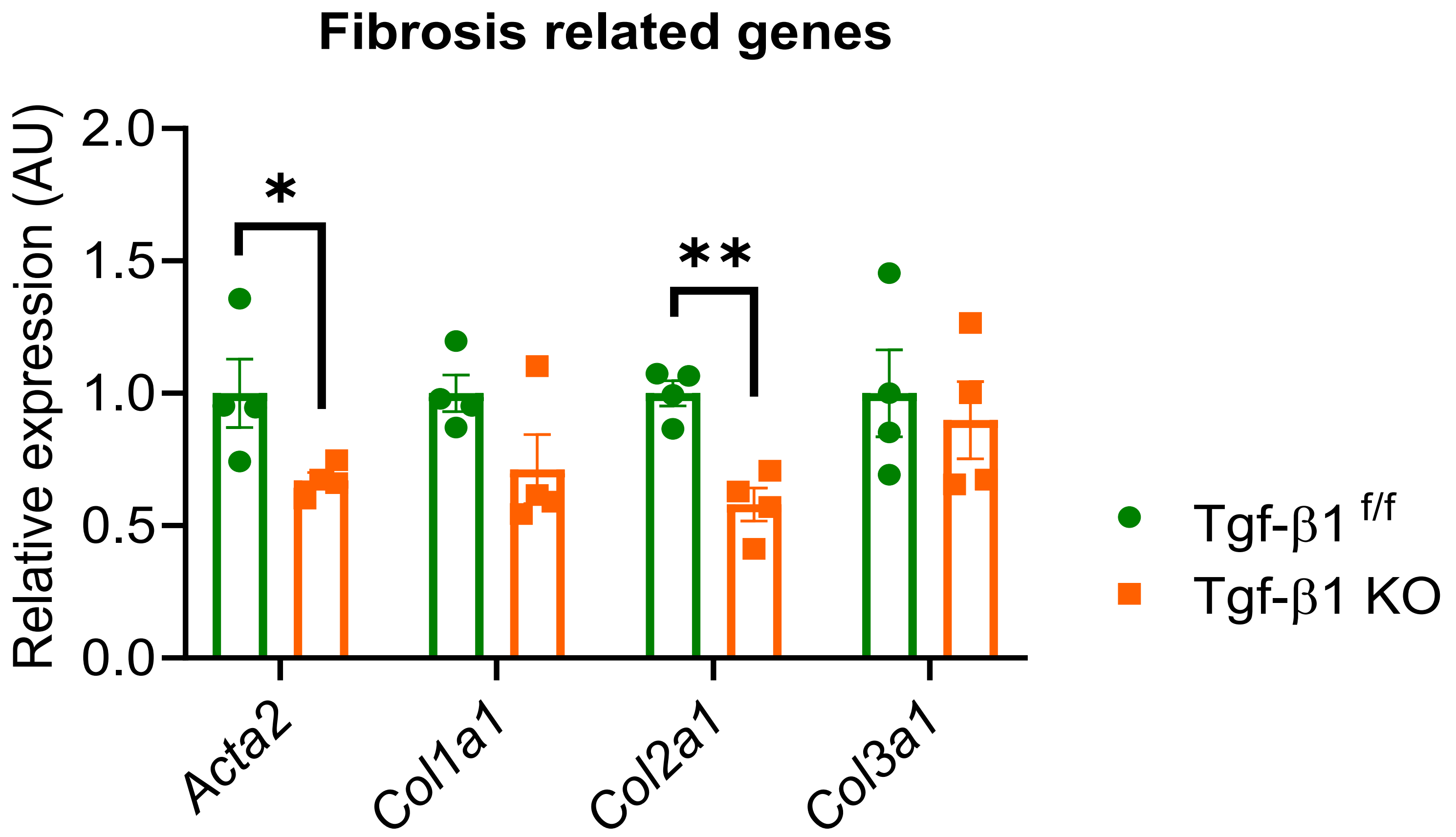

Figure S6: Fibrosis-related gene expressions in TA muscle (*n* = 4, 4). Data represent mean ± SEM. Statistical analysis was performed using a two-tailed unpaired *t*-test (*$p < 0.05$, **$p < 0.01$).

Figure S7

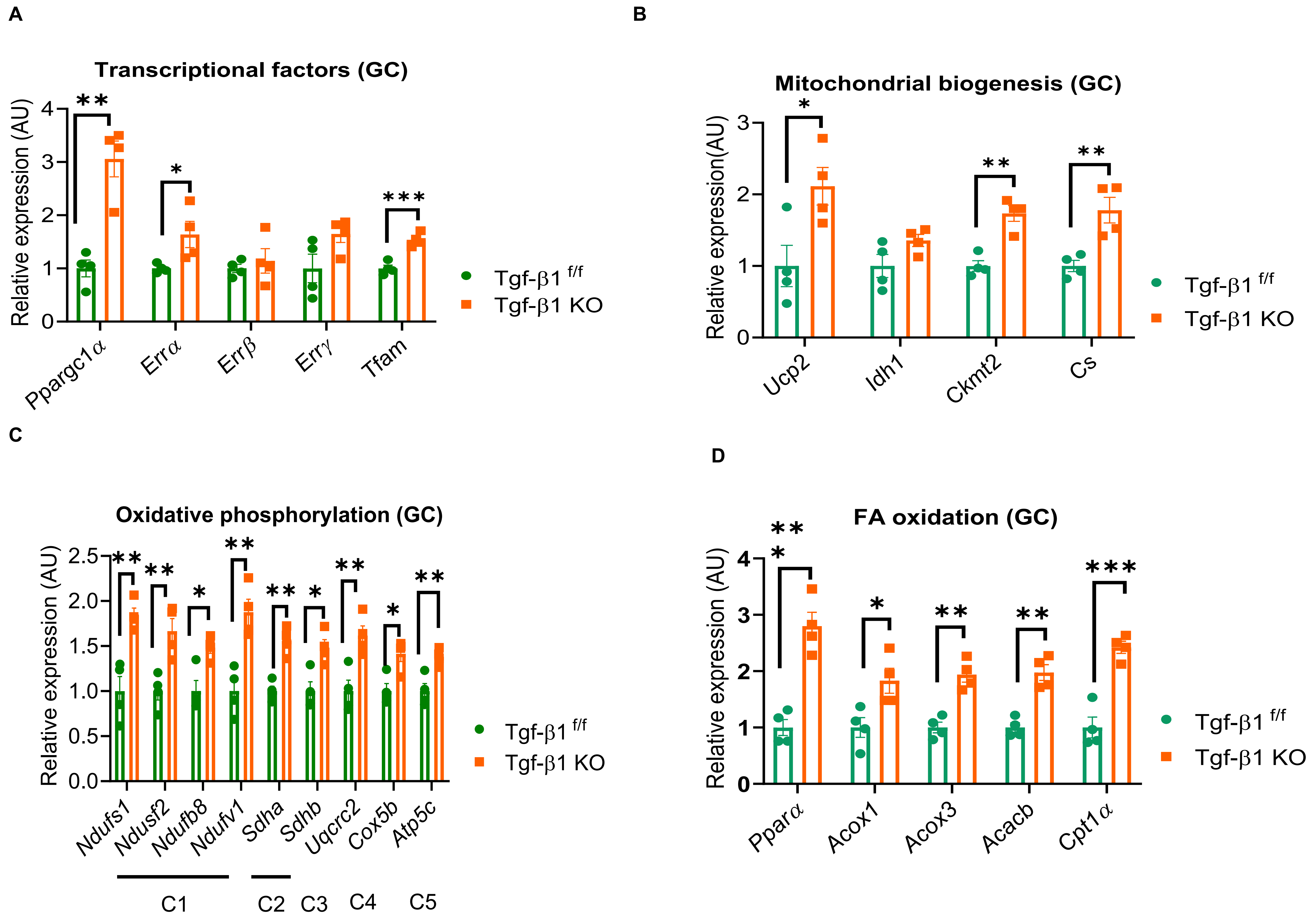

Figure S7: Deletion of CD206$^+$ M2 macrophage-specific Tgf-β1 stimulates mitochondrial biogenesis and FA oxidation in GC. (A) Relative mRNA expression of mitochondrial transcriptional factor–related genes in GC ($n = 4$, 4). (B) Relative mRNA expression of mitochondrial biogenesis–related genes in GC ($n = 4$, 4). (C) Relative mRNA expression of oxidative phosphorylation–related genes in GC ($n = 4$, 4). (D) Relative mRNA expression of FA oxidation–related genes in GC ($n = 4$, 4). Data represent mean ± SEM. Statistical analysis was performed using a two-tailed unpaired *t*-test (*$p < 0.05$, **$p < 0.01$, ***$p < 0.001$).

Figure S8

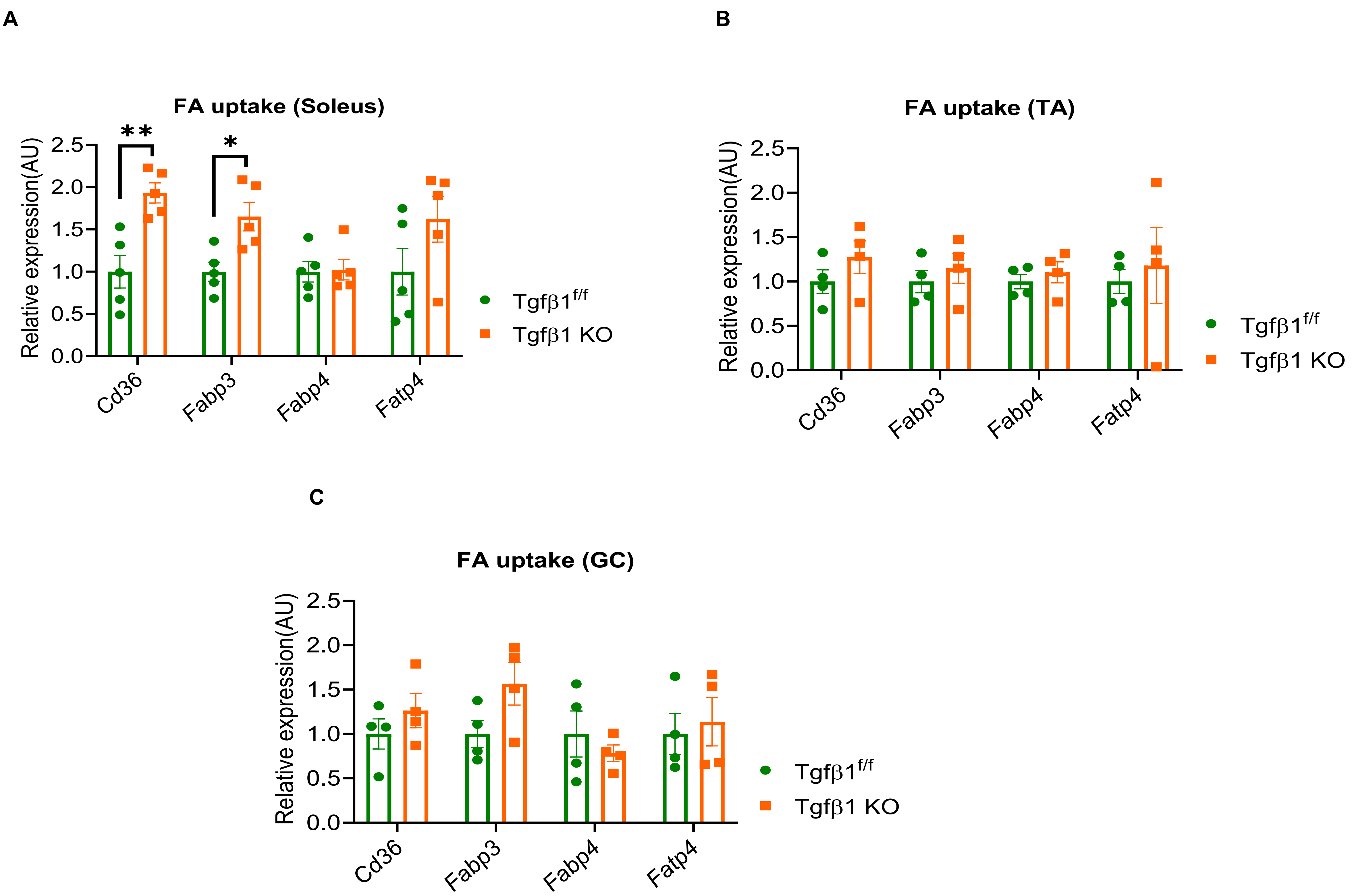

Figure S8: FA uptake-related gene expression in muscle. (A) Relative mRNA expression of FA uptake-related genes in soleus ($n = 5, 5$). (B) Relative mRNA expression of FA uptake-related genes in TA ($n = 4, 4$). (C) Relative mRNA expression of FA uptake-related genes in GC ($n = 4, 4$). Data represent mean ± SEM. Statistical analysis was performed using a two-tailed unpaired *t*-test (*$p < 0.05$, **$p < 0.01$).

Figure S9

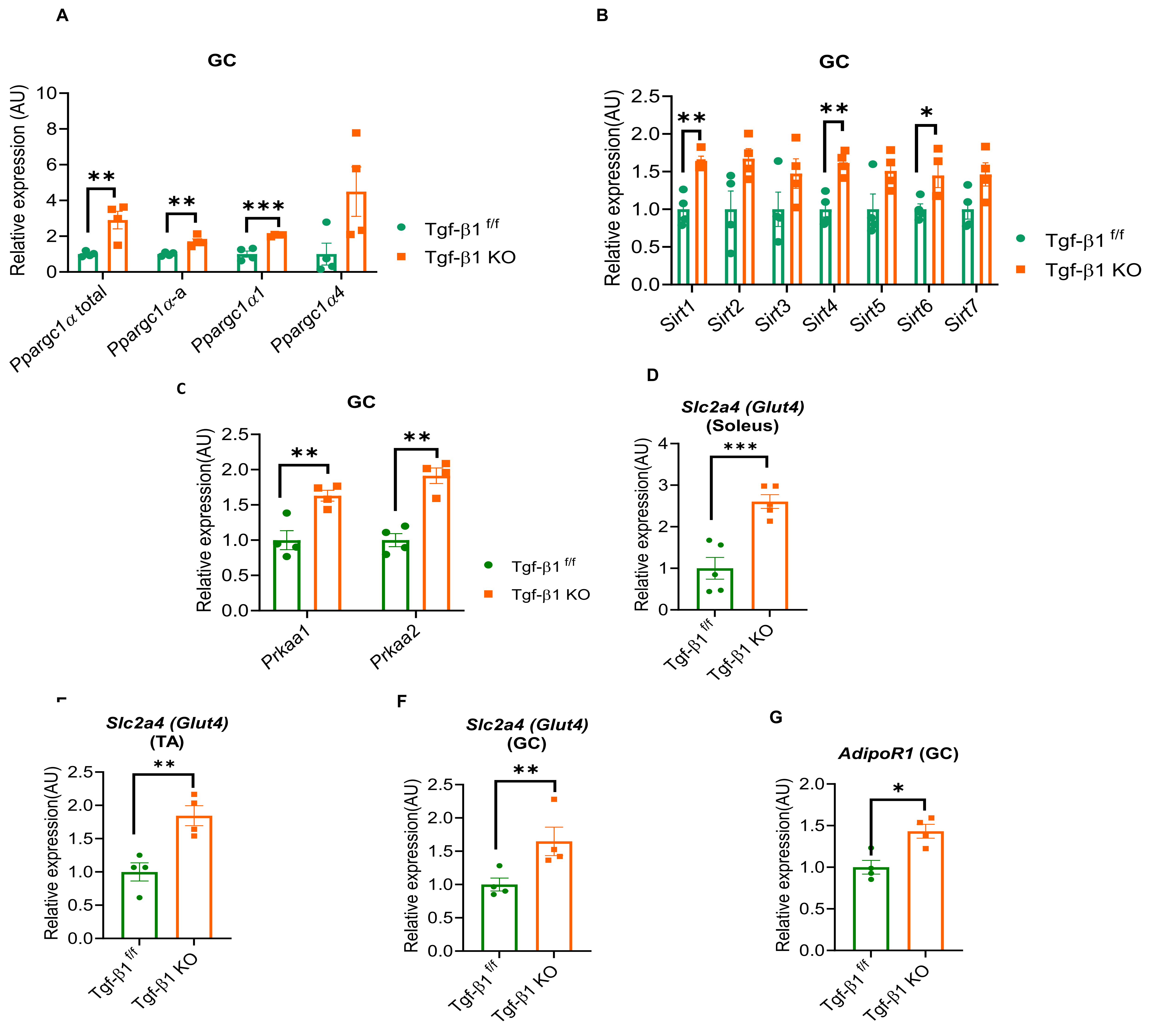

Figure S9: Deletion of CD206^{+} M2 macrophage-specific Tgf-β1-activated *Ppargc1α*, *Slc2a4* (*Glut4*) expression in skeletal muscle. (A) Relative mRNA expression of PGC1α-isoform genes in GC ($n = 4$, 4). (B) Relative mRNA expression of sirtuin-related genes in GC ($n = 4$, 4). (C) Relative mRNA expression of AMPKα-related genes in GC ($n = 4$, 4). (D) Relative mRNA expression of *Slc2a4* (*Glut4*) gene in soleus ($n = 5$, 5). (E) Relative mRNA expression of *Slc2a4* (*Glut4*) gene in TA ($n = 4$, 4). (F) Relative mRNA expression of *Slc2a4* (*Glut4*) gene in GC ($n = 4$, 4). (G) Relative mRNA expression of *AdipoR1* gene in GC ($n = 4$, 4). Data represent mean ± SEM. Statistical analysis was performed using a two-tailed unpaired *t*-test ($*p < 0.05$, $**p < 0.01$, $***p < 0.001$).

Supplementary References:

S1. Roh, E. and K.M. Choi, *Health Consequences of Sarcopenic Obesity: A Narrative Review.* Front Endocrinol (Lausanne), 2020. **11**: p. 332.

S2. Sousa, L.G.O., et al., *The effects of diet composition and chronic obesity on muscle growth and function.* J Appl Physiol (1985), 2021. **130**(1): p. 124-138.

S3. Londhe, P. and J.K. Davie, *Gamma interferon modulates myogenesis through the major histocompatibility complex class II transactivator, CIITA.* Mol Cell Biol, 2011. **31**(14): p. 2854-66.

S4. De Micheli, A.J., et al., *Single-Cell Analysis of the Muscle Stem Cell Hierarchy Identifies Heterotypic Communication Signals Involved in Skeletal Muscle Regeneration.* Cell Rep, 2020. **30**(10): p. 3583-3595.e5.

S5. Oprescu, S.N., et al., *Temporal Dynamics and Heterogeneity of Cell Populations during Skeletal Muscle Regeneration.* iScience, 2020. **23**(4): p. 100993.

S6. Tidball, J.G. and S.A. Villalta, *Regulatory interactions between muscle and the immune system during muscle regeneration.* Am J Physiol Regul Integr Comp Physiol, 2010. **298**(5): p. R1173-87.

S7. Mann, C.J., et al., *Aberrant repair and fibrosis development in skeletal muscle.* Skelet Muscle, 2011. **1**(1): p. 21.

S8. Contreras-Shannon, V., et al., *Fat accumulation with altered inflammation and regeneration in skeletal muscle of CCR2-/- mice following ischemic injury.* Am J Physiol Cell Physiol, 2007. **292**(2): p. C953-67.

S9. Perdiguero, E., et al., *p38/MKP-1-regulated AKT coordinates macrophage transitions and resolution of inflammation during tissue repair.* J Cell Biol, 2011. **195**(2): p. 307-22.

S10. Reidy, P.T., et al., *Aging-related effects of bed rest followed by eccentric exercise rehabilitation on skeletal muscle macrophages and insulin sensitivity.* Exp Gerontol, 2018. **107**: p. 37-49.

S11. Sorensen, J.R., et al., *An altered response in macrophage phenotype following damage in aged human skeletal muscle: implications for skeletal muscle repair.* Faseb j, 2019. **33**(9): p. 10353-10368.

S12. Tam, C.S., et al., *Low macrophage accumulation in skeletal muscle of obese type 2 diabetics and elderly subjects.* Obesity (Silver Spring), 2012. **20**(7): p. 1530-3.

S13. Wosczyna, M.N., et al., *Mesenchymal Stromal Cells Are Required for Regeneration and Homeostatic Maintenance of Skeletal Muscle.* Cell Rep, 2019. **27**(7): p. 2029-2035.e5.

S14. Scott, R.W., et al., *Hic1 Defines Quiescent Mesenchymal Progenitor Subpopulations with Distinct Functions and Fates in Skeletal Muscle Regeneration.* Cell Stem Cell, 2019. **25**(6): p. 797-813.e9.

S15. Bucala, R., et al., *Circulating fibrocytes define a new leukocyte subpopulation that mediates tissue repair.* Mol Med, 1994. **1**(1): p. 71-81.

S16. Inoue, A., et al., *Exercise restores muscle stem cell mobilization, regenerative capacity and muscle metabolic alterations via adiponectin/AdipoR1 activation in SAMP10 mice.* J Cachexia Sarcopenia Muscle, 2017. **8**(3): p. 370-385.

S17. Bilal, M., et al., *Fate of adipocyte progenitors during adipogenesis in mice fed a high-fat diet.* Mol Metab, 2021. **54**: p. 101328.

S18. Lagouge, M., et al., *Resveratrol improves mitochondrial function and protects against metabolic disease by activating SIRT1 and PGC-1alpha.* Cell, 2006. **127**(6): p. 1109-22.

S19. Bilal M., et al., *Tofogliflozin ameliorates cardiotoxin induced skeletal muscle injury and fibrosis in obesity*. Sci Rep. 2025 Oct 22;15(1):32633. doi: 10.1038/s41598-025-12734-9.

S20. Nishida, Y., et al., *Astaxanthin stimulates mitochondrial biogenesis in insulin resistant muscle via activation of AMPK pathway.* J Cachexia Sarcopenia Muscle, 2020. **11**(1): p. 241-258.

S21. Wosczyna, M.N., et al., *Multipotent progenitors resident in the skeletal muscle interstitium exhibit robust BMP-dependent osteogenic activity and mediate heterotopic ossification.* J Bone Miner Res, 2012. **27**(5): p. 1004-17.

S22. Vallecillo-García, P., et al., *Odd skipped-related 1 identifies a population of embryonic fibro-adipogenic progenitors regulating myogenesis during limb development.* Nat Commun, 2017. **8**(1): p. 1218.

S23. Jørgensen, S.B., E.A. Richter, and J.F. Wojtaszewski, *Role of AMPK in skeletal muscle metabolic regulation and adaptation in relation to exercise.* J Physiol, 2006. **574**(Pt 1): p. 17-31.

S24. Ratnayake, D., et al., *Macrophages provide a transient muscle stem cell niche via NAMPT secretion.* Nature, 2021. **591**(7849): p. 281-287.

S25. Shang, M., et al., *Macrophage-derived glutamine boosts satellite cells and muscle regeneration.* Nature, 2020. **587**(7835): p. 626-631.

S26. Aslam MR. *et al.,* "Deletion of fibro-adipogenic progenitors-specific follistatin impairs muscle function and accelerates skeletal muscle atrophy in obese mice". Mol Med. 2025 Nov 21;31(1):340. doi: 10.1186/s10020-025-01393-1.

S27. Zong, H., et al., *AMP kinase is required for mitochondrial biogenesis in skeletal muscle in response to chronic energy deprivation.* Proc Natl Acad Sci U S A, 2002. **99**(25): p. 15983-7.

S28. Chang, H.C. and L. Guarente, *SIRT1 and other sirtuins in metabolism.* Trends Endocrinol Metab, 2014. **25**(3): p. 138-45.

S29. Cantó, C., et al., *Interdependence of AMPK and SIRT1 for metabolic adaptation to fasting and exercise in skeletal muscle.* Cell Metab, 2010. **11**(3): p. 213-9

S30. Ji, L.L. and C. Kang, *Role of PGC-1α in sarcopenia: etiology and potential intervention - a mini-review.* Gerontology, 2015. **61**(2): p. 139-48.

S31. Samdani, P., et al., *A Comprehensive Inter-Tissue Crosstalk Analysis Underlying Progression and Control of Obesity and Diabetes.* Sci Rep, 2015. **5**: p. 12340.
