# Supplementary material for: Deletion of Tgf‐β1 From CD206+ M2 Macrophages Ameliorates Obesity‐Induced Suppression of Myogenesis and AMPK Phosphorylation in Skeletal Muscle
Source: J Cachexia Sarcopenia Muscle. 2026 Jun 18;17(3):e70322. doi: 10.1002/jcsm.70322 (PMC13280218; doi:10.1002/jcsm.70322)
Supplement: Supplementary file 2 — Table S1: Key resources table. Table S2: Primer list. [file JCSM-17-e70322-s002.pdf]

Table 1: KEY RESOURCES TABLE

REAGENT or RESOURCE	SOURCE	IDENTIFIER
Antibodies		
Anti-mouse CD206	Santa Cruz Biotechnology	Cat#Sc 58987
Anti-rabbit TGFβ1	Santa Cruz Biotechnology	Cat#Sc-146
Alexa Fluor® 555 conjugate goat anti-rabbit IgG (H+L)	Cell signaling	Cat#4413
Anti-mouse IgG Fab2 Alexa Fluor (R) 488	Molecular Probes	Cat#A11059
AMPKα	Cell signaling	Cat#5831P
p-AMPKα	Cell signaling	Cat#2535P
Akt	Cell signaling	Cat#9272S
P-Akt	Cell signaling	Cat#9271S
Acetyl-CoA (ACC)	Cell signaling	Cat#3676P
p-ACC	Cell signaling	Cat#11818P
PGC1α	Santa Cruz Biotechnology	Cat#sc13067
β-actin	Cell signaling	Cat#4967S
ECL ^{TM} Anti-Rabbit IgG	GE Healthcare	NA934V
**Chemicals, Peptides, and Recombinant Proteins**		
Tamoxifen	Sigma-Aldrich	Cat#T5648
Sunflower oil	Fujifilm Wako Chemicals	Cat#196-15265
Bovine serum albumin	Sigma-Aldrich	Cat#A8022-100G
BD Pharm lyse lysing buffer	BD Biosciences	Cat#555899
PBS (-)	Nacalai tesque	Code 14249-24
DAKO real antibody diluent	DAKO	Ref# S2022
Fetal bovine serum (FBS)	Gibco	Ref# 10437-028
Ethanol (99.5%)	Fujifilm WAKO Chemicals.	Cat#057-00456
4% Paraformaldehyde phosphate buffer solution	Fujifilm WAKO Chemicals.	Cat#163-20145
Direct PCR (Tail)	Viagen Biotech Incorporation	Cat#102-T
Proteinase K, recombinant PCR grade	Roche Diagnostics	Cat#03115828001
TKS GflexTM DNA polymerase	Takara	Cat# R060A
Ethidium bromide	Invitrogen	Cat#15585-011
Agarose S gel	Nippon Gene	Cat#312-01193
Mini-PROTEAN TGX^{TM} Precast Gels	Bio-Rad Laboratories	Cat#456-1086
**Critical Commercial Assays**		
Isogen	NIPPON GENE	Code#311-02501resourcesresources
RNeasy mini kit (250)	Qiagen	Cat#74106

Mouse Adiponectin ELISA kit	Proteintech	Cat#KE10044
TB Green Premix Ex Taq^{TM} II (Tli RNaseH Plus)	TAKARA	Cat#RR820A
TB Green Fast qPCR Mix	TAKARA	Cat# RR430A
New hematoxylin solution	Muto Pure chemicals Co, limited	Lot# 131209
New eosin solution	Muto Pure chemicals Co, limited	Lot# 131028
β-Mercaptoethanol	Gibco	Cat#21985-023
**Experimental Models: Organisms/Strains**		
CD206-CreER^{T2};Tgfβ1$^{f/f}$	Nawaz et al (2022, Nature Communication)	
**Software and Algorithms**		
Graph pad prism	Version 9	
**Other**		
Normal chow (NC) diet	CLEA, Japan	CE-2
High-fat diet (60% KCAL Fat)	Research Diets	Cat# D12492

Table 2: Primer list

Primer Name	**Sequence**	
	Forward	Reverse
Tgfβ1	AAGTTGGCATGGTAGCCCTT	GCCCTGGATACCAACTATTGC
Adiponectin	TGTTCCTCTTAATCCTGCCCA	CCAACCTGCACAAGTTCCCTT
AdipoR1	AGTGCATGGTGGGTACAACA	GCATCTCTGCCATCATTGTG
Ppargc1α	CCCTGCCATTGTTAAGACC	TGCTGCTGTTCCTGTTTTC
Ppargc1 α total_sp	TGATGTGAATGACTTGGATACAGACA	GCTCATTGTTGTACTGGTTGGATATG
Ppargc1 α -a	GGGACATGTGCAGCCAAGA	AAGAGGCTGGTCCTCACCAA
Ppargc1 α -1	GGACATGTGCAGCCAGACTCT	CACTTCAATCCACCCAGAAAGCT
Ppargc1 α -4	TCACACCAAACCCACAGAAA	CTGGAAGATATGGCACAT
Sirt1	CCAGACCCTCAAGCCATGTT	CTGTCCGGGATATATTTCCTTTGC
Sirt2	GGCTCAGGATTCAGACTCGG	GCTCGTCTAGAAGACGCTCC
Sirt3	ACACAATGTCGGGTTTCACA	CAGCTACATGCACGGTCTGT
Sirt4	CGGACGTCGTTTTCTTTGGG	CCTGTAACCAGAGTACACCTGC
Sirt5	ACAGGGCGGTTAAGAAGTCC	GGCCGAGTTTAACATGGAGA

Sirt6	ACCCACAAAACATGACCGCC	TTTGTCTAGCACGCAGGGTC
Sirt7	CCGTCGACCCAAACTCTACA	CTGCCACCGGTTGTAGACAG
AMPKα1	ACCAGGTCATCAGTACACCA	TCCTTTTCGTCCAACCTTCCA
AMPKα2	GGCAAAGTGAAGACTACCAGG	CTTCAACCCGCCCATGTTTG
Glut4	CAGTGTTCCAGTCACTCGCT	CAGCGCCTGAATCTTTTCTT
Pparγ	TTTTCCGAAGAACCATCCGATT	ATGGCATTGTGAGACATCCCC
Ndufs1	TGCAAATCCCTCGATTCTGTTAC	GCTTTCTCAATCTCTACCAGGC
Ndusf2	TCGTGCTGGAACTGAGTGGA	GGCCTGTTCATTACACATCATGG
Ndufb8	CCCGCTCCAGGTACAGATTA	GGTTGGTAAAGATCCGGTCTTC
Ndufv1	GTGCGGGTATCTGTGCGTT	GCTTTCTCAATCTCTACCAGGC
Sdha	GCTTGCGAGCTGCATTTGG	TGTGATCGGGTAGGAAAGAGC
Sdhb	TCTGGGTCCCATCGGTAAAT	GCCGTTCTCGGCAGAGTC
Uqcrc2	GATAACCCGTGGGATTGAAGC	TCTACAGTGTACGCCATGTTTTC
Cox5b	GGAAGACCCTAATCTAGTCCCG	GTTGGGGCATCGCTGACTC
Atp5c1	CCAGGAGACTGAAGTCCATCA	AGAACCTGTCCCATACACTCG
Errα	CAGCTGTACTCGATGCTCCC	AGCTCTCTACCCAAACGCCT
Errβ	TGAAGGAGCCGCAACTAGAG	GCTGGAACACCTGAGGGTAA
Errγ	TTGTACTTCTGCCGACCTCC	TGAGATCACAAAGCGCAGAC
Tfam	CAGGAGGCAAAGGATGATTC	CCAAGACTTCATTTCATTGTCG
Ucp2	TGGAAAATCGAGGGGATCGG	GGAGAAACGGGGACCTTCAA
Idh1	CTCTGCAGCATCTTTGGTGA	TGACACGAATCATTTGGGAA
Ckmt2	TTCGGCCAGTTAGCAACTTT	TTACACTTCTTGGCTGTGTGC
Cs	AAGGACGAGGCAGGATGAG	TGCAGCTGTAGCTCTCTCCC
Pparα	TTTTCCGAAGAACCATCCGATT	ATGGCATTGTGAGACATCCCC
Acox1	GGAATTTGGCATCGCAGACC	ACATGCCCAAGTGAAGGTCC
Acox3	GCTCGGTAGGCACTAAGAGG	CTTCTGAGAAACGGGGACAA
Acacb	TGTTCTCGGCCTCTCTTCAC	GAGGCTGCATTGAACACAAG
Cpt1α	TTGGAAGTCTCCCTCCTTCA	GCCCATGTTGTACAGCTTCC
Cd36	ACAGCTGCCTTCTGAAATGTGT GGA	TTTTCTACGTGGCCCGGTTCTAA TTCA
Fabp3	CCGTTCTTCTCGATGATGGT	CGGTACCTGGAAGCTAGTGG
Fabp4	AAATCACCGCAGACGACAGG	ATAACACATTCCACCACCAGC
Myh7	CTCAGGTGGCTCCGAGAAAG	TGGCTGAGCCTTGGATTCTC

Myh3	GACTCAGCCGACACCATGAG	CTCTGGCTTAACCACCAGGG
Myh4	TCTGGTAACACAAGAGGTGC	AAAAGGCTTGTTCTGGGCCT
Myh2	GCAAGAAGCAGATCCAGAAAC	GGTCTTCTTCTGTCTGGTAAGTA AGC
Myh1	GCAACAGGAGATTTCTGACCTC AC	CCAGAGATGCCTCTGCTTC
MyoG	GTGAATGCAACTCCCACAGC	CGCGAGCAAATGATCTCCTG
Pax7	CGGGTTCTGATTCCACATCT	CGACGAGGAAGGAGACAAGA
Myf5	GACGGCATGCCTGAATGTAAC	GCTGGACAAGCAATCCAAGC
Pdgfrα	AGCAGGCAGGGCTTCAACGG	ACACAGTCTGGCGTGCGTCC
Dpp4	AGTGAATACGTTCTGCGGCT	TGAAGACACCGTGGAAGGTT
Fst	GACAATGCCACATACGCCAG	GTTTCTTCCGAGATGGAGTTGC
Fstl1	GCCAGCTCCACAAAACACAT	GAGCACGATGTGGAAACGAT
alpha-SMA	CTCTCTTCCAGCCATCTTTCAT	TATAGGTGGTTTCGTGGATG
Acta2	GACGCTGCTCCAGCTATGT	CCAACCATTACTCCCTGATGT
Col1a1	GCTCCTCTTAGGGGCCACT	CCACGTCTCACCATTGGGG
Col3a1	CTGTAACATGGAAACTGGGGAA A	CCATAGCTGAACTGAAAACCACC
Collagen type Ⅱ	CCGGTATGTTTCGTGCAGCCAT CCT	GGCAATAGCAGGTTCACGTACA

Lead contact

Further information and requests for resources and reagents should be directed to and will be fulfilled by the Lead Contact, Kazuyuki Tobe (tobe@med.u-toyama.ac.jp)
